# Recent Updates on Viral Oncogenesis: Available Preventive and Therapeutic Entities

**DOI:** 10.1021/acs.molpharmaceut.2c01080

**Published:** 2023-07-24

**Authors:** Shivam Chowdhary, Rahul Deka, Kingshuk Panda, Rohit Kumar, Abhishikt David Solomon, Jimli Das, Supriya Kanoujiya, Ashish Kumar Gupta, Somya Sinha, Janne Ruokolainen, Kavindra Kumar Kesari, Piyush Kumar Gupta

**Affiliations:** 1 Department of Industrial Microbiology, Sam Higginbottom University of Agriculture, Technology and Sciences, Prayagraj 211007, Uttar Pradesh India; 2 Department of Bioengineering and Biotechnology, Birla Institute of Technology, Mesra, Ranchi 835215, Jharkhand, India; 3 Department of Applied Microbiology, Vellore Institute of Technology, Vellore 632014, Tamil Nadu, India; 4 Department of Life Sciences, Sharda School of Basic Sciences and Research, Sharda University, Greater Noida 201310, Uttar Pradesh, India; 5 Department of Molecular & Cellular Engineering, Sam Higginbottom University of Agriculture, Technology and Sciences, Prayagraj 211007, Uttar Pradesh, India; 6 Centre for Biotechnology and Bioinformatics, Dibrugarh University, Assam 786004, India; 7 School of Biotechnology, Jawaharlal Nehru University, New Delhi 110067, India; 8 Department of Biophysics, All India Institute of Medical Sciences, New Delhi 110029, India; 9 Department of Biotechnology, Graphic Era Deemed to Be University, Dehradun 248002, Uttarakhand, India; 10 Department of Applied Physics, School of Science, Aalto University, 02150 Espoo, Finland; 11 Division of Research and Development, Lovely Professional University, Phagwara 144411, Punjab, India; 12 Faculty of Health and Life Sciences, INTI International University, Nilai 71800, Malaysia

**Keywords:** Oncoviruses, Viral Oncogenesis, Antiviral Agents, Therapeutics, Malignancy

## Abstract

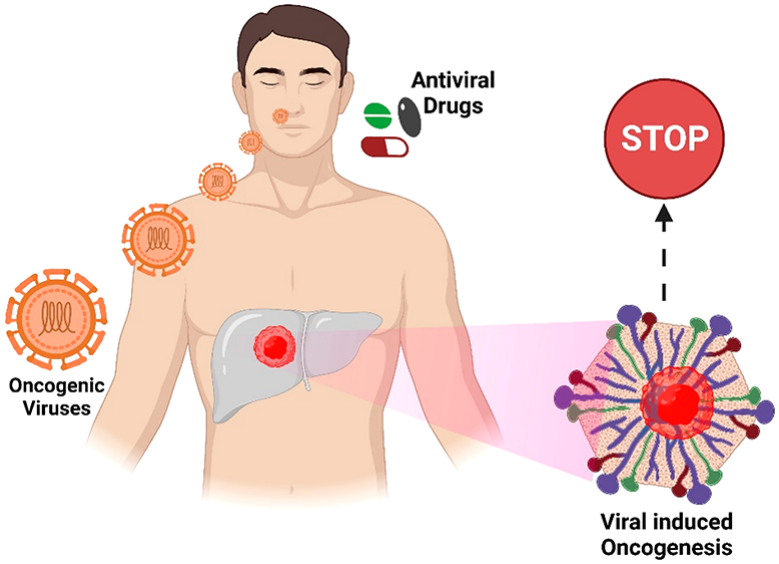

Human viral oncogenesis is a complex phenomenon and a major contributor to the global cancer burden. Several recent findings revealed cellular and molecular pathways that promote the development and initiation of malignancy when viruses cause an infection. Even, antiviral treatment has become an approach to eliminate the viral infections and prevent the activation of oncogenesis. Therefore, for a better understanding, the molecular pathogenesis of various oncogenic viruses like, hepatitis virus, human immunodeficiency viral (HIV), human papillomavirus (HPV), herpes simplex virus (HSV), and Epstein-Barr virus (EBV), could be explored, especially, to expand many potent antivirals that may escalate the apoptosis of infected malignant cells while sparing normal and healthy ones. Moreover, contemporary therapies, such as engineered antibodies antiviral agents targeting signaling pathways and cell biomarkers, could inhibit viral oncogenesis. This review elaborates the recent advancements in both natural and synthetic antivirals to control viral oncogenesis. The study also highlights the challenges and future perspectives of using antivirals in viral oncogenesis.

## 1 Introduction

The human body is phenomenal specimen with an intricate signaling pathway network. The body has its own defense mechanism to fight against viruses and/or disease-causing agents. Suppressing the body’s defense mechanisms, cells can start behaving abnormally and divide in a malignant mode. This may lead to cancer in the human body. The formation of cancer malignancy does not rely on one aspect. It involves a wide range of interconnected factors such as genetic alterations, environmental exposures, and lifestyle factors.^1^ Although, a class of cancer-causing viruses may develop cancers in humans due to oncogenic virus infections.^2^ Among all types of cancer, the oncogenic virus triggers the formation of 10–20% of human cancers.^3,4^ Viruses are known to replicate only when they control the host mechanisms in their favor. The characteristic feature of oncogenic viruses is the induction of viral tumorigenesis that helps in the propagation of malignancy from infected cells to healthy cells.^2^ Oncogenic viruses can stimulate cancer formation with the help of several cofactors, such as persistent inflammation, suppression of cancer-specific immune agents, and involvement of cancer-causing mutagens.^5^ Inflammatory signals released by infected cells act as a gateway for viral tumorigenesis. White blood cells of the immune system get alerted and help to clear the infected cells. However, with persistent or chronic inflammation, clearing and repairing such cells remain unchecked, thus leading to severe DNA damage with multiple mutational events that pave the way for cancer.^6^ Following inflammation, oncogenic viruses can counter the protective action of p53, retinoblastoma tumor-suppressing pathways.^7^ The addition of environmental exposure to mutagens accelerates the process of viral tumorigenesis.

**Figure 1:**
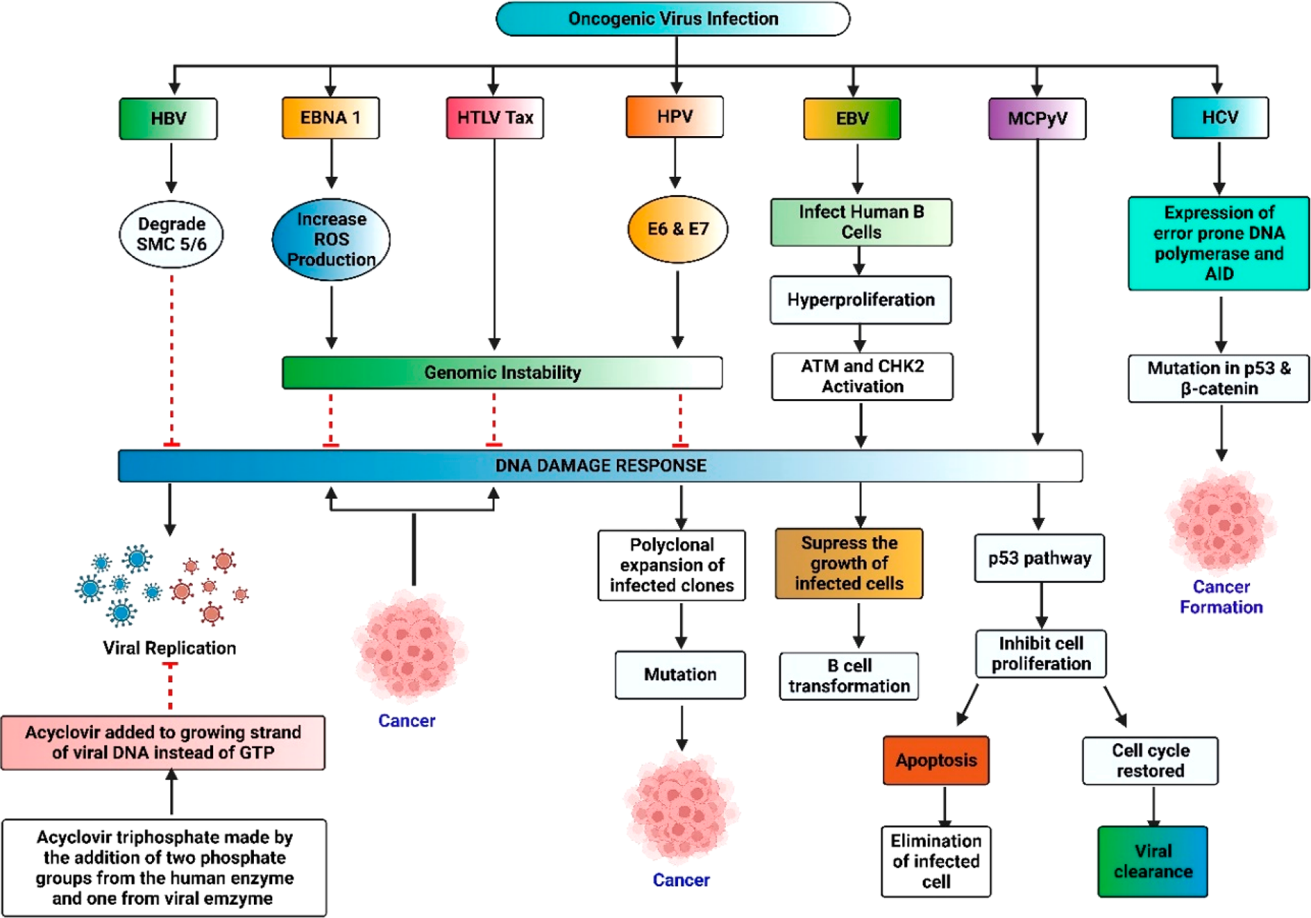
Various oncogenic viral strains have taken various pathways to infect the host organism, leading to cancerous proliferation (Created in Biorender).

Viral oncogenes can be grouped into two main categories: first replicating their genetic material (oncogenic DNA viruses) and second, replicating their genetic information.^8^ Oncogenic DNA viruses include Epstein–Barr virus (EPV), hepatitis B virus (HBV), human papillomavirus (HPV), Merkel cell polyomavirus (MCPyV), and human herpesvirus-8 (HHV-8). Additionally, oncogenic RNA viruses encompass diseases like hepatitis C (HCV) and human T-cell lymphotropic virus type 1 (HTLV-1) (Figure 1). The characteristic feature of oncogenic DNA viruses is to activate the DNA damage response pathway (DDR). DDR is crucial for DNA repairing of damaged cells induced by viral tumorigenesis.^8^ Their inactivation delays the cell cycle and helps the virus to skip the apoptotic pathways, thus favoring the propagation of oncogenic DNA viruses.^9^ The need to prevent the spread of viral tumorigenesis has become a matter of deep concern in recent years. Antiviral therapeutics and viral vaccines are able to specify the spread of malignant cells and induce localized killing of malignant cells. Along with the development of novel antiviral therapies, there is an evident change in the pathogenicity pattern of oncogenic viruses that needs to be investigated simultaneously.

This review discusses the major discoveries in recent times concerning antiviral therapies along with the analysis of viral tumorigenic pathways. Developing novel antiviral therapeutics detrimental to preventing the spread of viral-induced cancers.

## 2 Viruses Associated with Oncogenesis

### 2.1 Hepatitis B Virus (HBV)

Studies over the past few decades revealed that HBV infection has been proven to promote disease progression via various mechanisms. HBV is an epidemic viral disease prevalent in many regions across the globe, with varying infection rates of 10–40% in New Zealand and Pacific Island countries like Fiji to 0.1% in the United States. HBV infection occurs more prominently in males with age group of 25–45-year. The prevalence percentage is recorded to be more in the urban areas of the United States. A similar age band of 25–45-year-old males are reported to be infected with HBV in the European subcontinent. Males in the 15–25 age band are also reported to be infected by HBV. Reports on infection criteria have suggested that the incidence rate of HBV is lower in females, with a caseload of as much as 0.58 compared to 1.33 cases per 100,000 in the population settings. The prevalence of hepatitis B surface antigen (HBsAg) determines the endemicity of HBV globally. The high endemic locations accounting for almost 50% of HBV-related cases belong to the African subcontinent, Asian subcontinent, the Great Amazon region, and part of Middle Eastern nations wherein the HBsAg rate is recorded to be at a whopping ≥ 8%. The low endemic rate locations, accounting for 10–12% of HBV-related cases, belong to the European subcontinent, the United States, and Australia, wherein the HBsAg rate is recorded as low as < 1%.^10,11^ Furthermore, this causes hepatocytes to become cancerous over time. Several variables have been implicated in the etiology of HBV-associated HCC, including integrating HBV genes into host cells, causing genomic instability due to mutation and activating signaling pathways promoting malignancy. Studies have shown that the hepatitis B virus is closely related to retroviruses and consists of a compact, partially double-stranded DNA molecule.^12^ The genome size is estimated to be around 3.2 kilobase pairs. Its framework is similar to the hepatotropic lineage of DNA viruses, classified in the genus Orthohepadnavirus and the family hepadnaviridae.

Based on the sequenced analogy of HBV, it is classified into 8 genotypes, and each genotype possesses different topographic dissemination. An infected serum consists of 3 virion types that can be visualized under an electron microscope. Among them, 2 virion types have small globular arrangements with filaments of different lengths with a width of 22 nm and a diameter of 20 nm.^13^ The filaments and spheres of the virions consist of the hepatitis B surface (HBsAg) as antigen and host-originated lipids. Still, they do not contain viral nucleic acid, so they are non-infectious.^14^ The third virion type and Dane particles (infectious HBV virus particles) have a spherically double-layered structure with a diameter of 42 nm and comprise a lipid coating containing HBsAg, which encloses the polymerase complex encoded by the virus and the viral DNA. The viral polymerase is bonded covalently to the 5′ terminus of the strand, which is negatively charged.^15^

The ORF (open reading frame) of the regulatory protein HBx or PreS2 can be transcribed and maintained in most of the unified subviral HBV genomes.^16^ The HBx gene is a tiny peptide (17 KDa) preserved in hepatitis viral strains of mammals and present at a minimum level during acute and chronic hepatitis. Recently, DHBV (duck hepatitis B virus) was recognized as a regulative protein similar to HBx^17^ where HBx has been known as a crucial viral protein in the carcinogen process related to HBV,^18,19^ which could significantly affect the HBx target. Knowing about the possible role of HBx in viral malignancy, the research shifted its focus to HBX’s intervention with a signal transduction cascade that impacts cell cycle regulation, proliferation, or apoptosis. However, it should be recalled that the overexpression of HBx stimulates those conditions which are different from the infected cells, reflecting the overall genetic makeup. In particular, HBx can promote cell proliferation, which has been fully confirmed.^20^

On the contrary, the transport of the entire HBV genome containing the regulatory proteins HBx and PreS2 tends to inhibit the progression of the cell cycle.^21^ There are reports about the obstruction of HBx on protein kinase C signaling. The inspection of the protein kinase C (PKC)/HBx interaction is one example of evidence that targeted the interference of HBx on the signal cascade and correlated it with the putative effect of HBx on HBV-related oncogenesis. Some studies indicate that HBx does not affect PKC activity, nor that PKC is important for HBx-dependent transcriptional instigation.^22^ In contrast, there is also evidence describing the dependence of HBx on PKC stimulation, which has been satisfied by an increased level of DAG in HBx-processing cells. This implies that PKC is crucial in HBx-dependent stimulation of NF-kB or AP1.^23^ A persuasive aspect of HBx-dependent PKC stimulation that is decisive model of HBx and can be derived from HBV-dependent oncogenesis.

According to the two-step malignant model,^24,25^ HBx-dependent PKC activation can play a role similar to that of a tumor promoter, regardless of whether it acts similarly to a tumor promoter.^24,25^ Experiments in transgenic mice showed strong evidence that HBx may function like a tumor promoter. Compared with wild-type control animals, the brilliance of HBx transgenes or disclosure of these transgenes to mutagens (diethylnitrosamine) results in a significant elevation in preneoplastic contusions.^26^ The tumor promoter-like role of HBx is not necessarily needed to stimulate PKC. Pathways such as compelling the cRaf1MEK/MAP2 kinase cascade (mitogen-activated protein kinase 2) can also achieve this activity. HBx was first found to elevate Ras/GTP complex production, resulting in the activation of the cRaf1 signal transfer cascade.^24,25^ That is why more information has been gathered to clarify the obstruction of HBx in the Ras upstream signal cascade. The other step is observing that Src is vital in HBx-forming cells. It was subsequently observed that HBx could activate the cytosolic Ca^2+^-dependent tyrosine kinase 2 Praline (Pyk).^27^

### 2.2 Human Papillomavirus (HPV)

The current data show that HPV infection and accompanying disorders are more common in developing nations. The global incidence rates of HPV in 2007, 2010, and 2019 were recorded as 10.4%, 11.7%, and 9.9%, respectively. The caseload of HPV is recorded to be highest in Asia, of which 57.7% from the Central and 44.4% from the East and South Asian subcontinent were HPV carriers. The Sub-Saharan Africa basin has an HPV caseload of 24%, of which the Eastern and Southern flanks consist of around 33% and 43% HPV woman carriers, respectively. The developed nations, mainly the European subcontinent, show a low caseload of around 4%. However, the Eastern European region shows a slightly higher caseload of HPV of around 22%. Reports suggest that females with the age group ≤ 25 years are infected by HPV prominently in the European and Asian subcontinent. Also, another study reported that females from the Eastern and Western flanks of Africa and the Central and Southern flanks of America showed higher HPV prevalence rates above the 45-year age group. Concerning the male population, HPV is sexually transmitted, and incidence rates for homosexual and HIV-positive men are higher than those for heterosexual men. The African male population constitutes the highest HPV incidence rate of around 18% per year, whereas the Asian male population presents a caseload of HPV of around 4% per year.^28,29^

The various factors such as HPV genotype, geographical conditions, population-based studies, and anatomical site sampling may impact the incidence and prevalence of these disorders. The nature portfolio states that human papillomavirus (HPV) is an infectious agent belonging to the papillomaviridae family. Its members are tropic for the skin epithelium and mucosal epithelium. More than 120 types of HPV are there, and HPV16 and HPV18 are firmly related to cervical cancer.^30^ Papillomaviruses are epitheliotropic, nonenveloped, double-stranded DNA viruses that cause infection in the mucosal and cutaneous epithelia in a species-specific way in a wide range of higher vertebrates and cause distortion of cellular metabolism leading to proliferation. It comprises various viruses that are capable of infecting animals and humans. Its emergence seems to come from the conversion in the epithelium of the ancestral host, as the initial reptiles appeared about 350 million years back. With the evolving time, they got advanced with their specific hosts, having almost no cross-species shift. They are now found in birds, mammals, marsupials, and reptiles, except amphibians or lower phylogenetic orders.^31^

The transmission of HPV is carried out sexually, but it has no penetration requirement. Genital skin contact is a conventional way of transmission. There are different types of HPV, many of which are harmless. The infection generally clears without any interference after a few months. It is the most common viral infection of the reproductive region. Most sexually active women and men get infected at some point, and some might get repeated infections. The climax time of the infection is soon after sexual activity. A small number of infections within a certain category of HPV can continue and turn into cervical malignancy. In terms of HPV-linked infections, the most common is cervical cancer. Certain HPV infections can cause carcinoma of the anus, oropharynx, vagina, vulva, and penis, which can be prevented by early treatment strategies.

The noncancerous group of papillomavirus (especially categories 6 and 11) can potentially cause genital warts and respiratory papillomatosis (a medical condition in which tumors grow in the airways that enter the lungs from the mouth and nose). Despite rarely leading to fatality, they can give rise to serious illnesses like condylomata acuminata, a very usual and highly contagious infection that can affect sexual life.^32^ Papillomavirus fragments have a common unenveloped icosahedral formation (50–60 nm in diameter), and its genome contains a double-stranded loop (episome) of approximately 8000 base pairs, containing 8 or 9 ORFs. Although the number of genes are limited and has a small genome size, the amount of ciphered proteins is much higher because gene expression requires various promoters and composite splicing patterns.^33^ The fine arrangements show^34^ that the virus coat contains 360 L1 protein molecules in 72 capsomeres. Each capsomere comprises five L1 molecules with a β-jellyroll nucleus similar to other icosahedral viruses.^35^

The interactions between the capsomeres require Late 1 protein, which stretches outward to the neighboring capsomeres and connects at their base by disulfide bridges.^36^ HPV also contains an irregular number of L2 molecules, besides the N-terminal 120 amino acids, which are not entirely uncovered on the virion surface.^37^ At the time of infection, L2 can bind to the extracellular fluid matrix and is separated by furin during infection.^38^ The surface of the L1 area, which is exposed, mainly contains a chain of the hypervariable amino acid coil, which differentiates between various kinds of papillomaviruses in feedback for selective immune constraint from the host. Antibodies against one kind of HPV have a small binding to distant types, and this has pragmatic implications for recent preventive vaccines that provide restricted cross-protection.

The viral genome also enciphers regulatory proteins that activate cell cycle entry and cell propagation, the proteins that moderate replication of the viral genome, virus association, and possibly influential virus discharge and escalation. Despite numerous genes being involved in a virus’s premature regions, the L2 gene out-turns, having an early key role in the transmission of the viral genome into the cell and playing a part in coordinating proper genomic loading (along with E2).^39^ The orderly expression of viral gene output that produces viral particles is altered in HPV-associated neoplasia.^40^ In cervix diseases, the expression levels of E6 and E7 are believed to increase from CIN1 to CIN3. Shifting the expression of these genes is the basis for different tumor phenotypes. CIN1 lesions generally support the full life cycle of HPV.^41^ It is believed that the increased activity of E6 and E7 occurs in high-threat HPV infections and is found to be the basis for the CIN2+ organization (phenotype), making cells prone to accumulating genetic errors, ultimately leading to cancer.^42^ Although many mechanisms will influence the persistence of HPV infection, the E6 and E7 proteins are essential and adequate for HPV-mediated tumorigenesis. In all human papillomavirus cases, the E6 and E7 proteins interact with many cellular proteins, and it is not easy to distinguish the definite reciprocal action that makes the E6 or E7 proteins carcinogenic.^2,43^ All papillomaviruses drive cell growth in the top layer of the epithelium to encourage the amplification of viral DNA. Yet, malignant HPV promotes cell cycle access and inactivates cell cycle barriers in the bottom layer of the affected epithelium.^44^ Compared to terminally superior differentiated cells, the emerging genetic instability in these growing cells has more serious repercussions. The essential functioning of HPV oncogenes is immunity-shifting, E7-interceding degeneration of pRB group members, E6-mediated degeneration of PDZ and p53 binding domain proteins, and telomerase-mediated elevation by E6.^2,43^ In contrast, β-HPV proteins E6 and E7 act as cofactors by inhibiting UV-induced DNA impaired repair and cell cycle arrest^45^ but are unnecessary to maintain tumor phenotype.

### 2.3 Kaposi-Sarcoma Associated Herpesvirus (KSHV)/Human Herpesvirus-8 (HHV-8)

KSHV became one of the well-recognized carcinogenic infectious agents in humans identified by the International Agency for Research on Cancer (IARC) after epidemiological and molecular investigations showed the link between KSHV and Kaposi sarcoma.^46^ HHV-8 viral strains are divided into A, B, C, D, E, and, most recently, N subtypes. The HHV-8 caseload in European countries is influenced by the A and C subtypes, and the B subtype influences African nations. HHV-8 D subtype influences populations belonging to Polynesian and Australian origins. Most recently, the N subtype has been identified as an HHV-8 influencer in the South African subcontinent.^47^

The human herpesvirus-8, or Kaposi’s sarcoma herpesvirus, resides in the DNA herpes virus family.^48^ Gamma herpesviruses are lymphotropic viruses that typically replicate in epithelial cells, such as blood vessels, organs, and skin. These viruses can set up a quiescent state for life in the host cell. When HHV-8 enters the incubation period in the vascular endothelium and B lymphocytes, it will experience a duration of lysis and replication intermittently, mainly under favorable conditions such as immunodeficiency, malnutrition, and solid organ transplantation. It can cause Kaposi’s sarcoma (a malignant vascular condition) and B-cell lymphoproliferative diseases like Multicentric Castleman disease (MCD) and primary exudative lymphoma (PEL). Although these tumors can happen without HIV coinfection, there is a possibility that people with the infection of HIV are significantly more probable to spread HHV-8-related malignancies. In rare instances, Kaposi sarcoma is detected in patients without AIDS and is generally found in elderly Mediterranean men or induced immunosuppressed patients, including transplant populations.^3,9,49^

KSHV is a large enveloped extended, double-stranded DNA virus and belongs to the subfamily of gamma herpesvirus and genus-rhadinovirus. It has an icosahedral capsid and an outer skin containing protein and RNA. It produces various proteins that validate the virus to conceal host immunity, replicate in the nucleus, and have a potent carcinogenic response. These proteins interact with several metabolic pathways in the cell, along with NF-kB (nuclear factor kappa-B), PI3K (phosphoinositide 3-kinase), RTA (replication and transcription activator), JAK/STAT (Janus signal/kinase transduction and transcription activator), and also MAPK (mitochondrial activated protein kinase). One of the most expressed proteins is the incubation period accessory nuclear antigen 1 (LANA), which is essential for the incubation period and tumorigenesis. Furthermore, cyclins of the virus have been noted to impede tumor suppressor genes (p53 and RB (retinoblastoma)) to hinder apoptosis by regulating the cell cycle.^3,50,51^

Regardless of the analogy between the viruses and their related tumors, the specific operation and activities of proteins related to virology and tumor-related aspects seem quite divergent. These proteins are believed to play a crucial role in virus biology and are mainly associated with the pathogenesis of viruses.^52^ The discovery of the vCCL 1–3, a viral chemokine, and the evidence of its pro-angiogenic activity in probing structure indicates its presumed role in immune elusion throughout the production of HHV-8, and these proteins can also cause diseases.^53^ The VGPCR and homologous of the chemokine receptor can be promoted to uphold the propagation of Kaposi sarcoma lesions and are found to induce suspected types of angiogenic cell cytokines.^54^ The ORF of HHV-8 K1 encodes the constructively active membrane receptors, along with K15.^55,56^ Although v-cytokines, K1, and VGPCR are expressed primarily or only during generative lytic replication, any contribution to oncogenic pathogenesis may be conciliated by paracrine signals.^57^

There is a piece of ample information suggesting that cytokine-mediated paracrine signal transmission plays a part in Kaposi Sarcoma and B cell proliferation, which can be affected through this pathway. HHV-8 specifies many proteins that were not found previously in gamma herpes viruses. In addition to the possible aspect of these viral proteins in the etiology of HHV-8, the role of some of these ″unique″ viral products in viral mechanisms has only recently received attention.^52^ Classical oncogenes and tumor suppressor activity are mediated by autocrine genes, and viral genes expressed in the latent period act as potential factors for malignancy. Among them, HHV-8 is primarily delayed nuclear-related antigen LANA, which describes the necessary replication and genetic makeup separation activities in splitting cells. It affects various host pathways to aid cell existence and growth. These activities are related to malignant transformation.^58^

### 2.4 Epstein–Barr Virus (EBV)/Human Herpes Virus 4 (HHV-4)

EBV strains exist in Type 1 and 2 forms, exhibiting varied occurrences throughout the globe. China and most European, American, and South Asian countries exhibit a higher prevalence of Type 1 EBV. Parts of the African subcontinent are prominent in prevalence rates concerning Type 2 EBV. In the case of head and neck squamous cell carcinoma (HNSCC), a coinfection pattern of EBV and HPV is observed. Biological testing in Taiwan and Morocco reported that the coinfected viral load for EPV/HPV in HNSCC stands at 48%. Similarly, testing carried out in Japan reported around 22% for the coinfected viral load, wherein no such viral load was observed in testing carried out in Greece and Denmark.^59^

Moreover, new insights into the mechanisms of EBV-infected cells’ malignant transformation, including somatic mutations and epigenetic modifications, their impact on the microenvironment, and the outcome of individual immune signatures related to the functional status of the immune system and the immune escaping approaches have been reported in recent years. The Epstein–Barr virus or Human Herpesvirus 4, which falls under the Gamma herpes virus, is an extended double-stranded DNA herpes virus with a length of approximately 172 kb.^60^ EBV spreads widely via close contact between asymptomatic EBV-infected and susceptible people who excrete the virus and are found frequently among adolescents and young adults.^61^ On the statistical scale, by the age of 5, antibodies against this virus have been found in approximately 50% of the population, and about 12% are vulnerable to adults who will develop antibodies in protection against the virus; half of the adults will thrive with mononucleosis disease.^62^ The EBV uses a dual strategy to ensure that many cells are infected to maintain the incubation period in the body.

On the contrary, viruses can propel infected cells into the cell cycle and proliferate, significantly increasing the number of cells carrying viral genomes. The virus replicates and releases infectious virus particles and can trigger a new round of infection. Therefore, the life cycle of a virus consists of at least three stages: i) the amplification of the infected cells that maintain the viral genome in a free state, ii) the establishment of the incubation period in the organism, and iii) the reactivation, replication, and synthesis of viral offspring. The incubation period of EBV (the time between the early infection and the onset of manifestation of symptoms) is unusually extended. It generally takes 4 to 7 weeks for the symptoms to appear.^63^ The most common sign of primary EBV infection is acute infectious mononucleosis, an auto-limiting clinical syndrome commonly affecting adolescents and young adults. Typical symptoms include malaise, fever, sore throat, fatigue, and systemic adenopathy.^64^

B-cell lymphoproliferative diseases that are closely related to EBV include Burkitt’s lymphoma (BL),^65^ Hodgkin’s lymphoma (HL),^66^ and post-transplant lymphoproliferative disease (PTLD).^67^ Many T-cell lymphoproliferative illnesses have been linked to the Epstein–Barr virus. These include cutaneous T-cell lymphoma, angioimmunoblastic T-cell lymphoma, NK/T cell extranodal sinus lymphoma, invasive leukemia/NK cell lymphoma, and peripheral T-cell lymphoma.^67,68^ Gastric cancer and nasopharyngeal malignancy are two examples of epithelial malignancies linked to EBV.^69^ Following the entry into B cells, viral DNA undergoes circularization by joining terminal repeats before being nucleosomalized, structured, and packed into micro-chromosomal structures known as episomes. Regulation of the switch from cleavage to possible replication requires post-translational changes in episomes. EBV latency schemes, namely, latency 1^st^, 2^nd^, and 3^rd^, have been established based on the latency gene articulation pattern in general B cells. Latency gene articulation is not restricted to the latent phase 3^rd^, only viable in an immunosuppressive state because the latent proteins are known to be highly immunogenic. Delay 2^nd^ latency is limited to EBER, EBNA-1, and LMP.^70^ Finally, according to the distinct phases of the cell cycle, there is no gene product expression in incubation period zero, or only EBNA-1 is indicated in the incubation period 1^st^.^71^ Latent phase 3^rd^ tumors develop under situations of disabled T cell immunity, like the infection of HIV or a transplantation-associated state of immunosuppression.^70^ EBV-linked giant B-cell and immunoblastic lymphomas frequently replace the incubation span program 3rd, independent of host immune activity.^72^ In latency 2^nd^ tumors, like nasopharyngeal tumefaction, NK/T cell lymphoma, and Hodgkin’s lymphoma, LMP1-mediated activation of the JAK/STAT and PI3K/AKT pathways have been inspected as main carcinogenic events.^70^ Finally, it is to be resolute whether EBV is the motivating force for the incubation period of 1^st^ tumors (like Burkitt Lymphoma). Most EBV-instigated tumor transformation mechanisms need several latent proteins, but EBNA-1 is generally considered the only viral protein expressed in the latent phase of 1^st^ cancer.^73^

EBV plays a supporting part in Burkitt lymphoma because of the integral activation of a major carcinogenic event c-myc; in all BL cases, EBV states.^74^ In the contrary, several reports have concluded that a small segment of these tumors have an extensive gene expression outline compared to those previously known, supporting the Epstein–Barr virus as a reason for incubation phase 1^st^ carcinoma. The main oncogenic protein of EBV is commonly considered to be the LMP1 and is crucial for transforming reclining primary B cells into growing lymphoblasts.^70^ LMP1 is a transmembrane protein that functions as a fundamental initiating CD40 receptor, which leads through the signaling pathways via downstream stimulation and participates in the differentiation of memory B cells and antiapoptotic protein expression.^70^ The NF-κB, PI3K/AKT, MAPK/ERK, Notch, and JAK/STAT are the downstream signaling pathways important in the oncogenesis induced by EBV.^75^ JAK/STAT and PI3K/AKT pathways are imperative in EBV-induced carcinogenesis.^68,70^ The incitement of the pathways PI3K/AKT and JAK/STAT contributes to the attributes of cancer, such as the elevated antiapoptosis, genomic vulnerability, unlimited replication potential, reorganization of intensive metabolism, and inflammation promotion of tumor, metastasis, and tissue seizure.^76^

Moreover, LMP1 induces genome disequilibrium by impeding DNA repair mechanisms and inhibiting DNA impairment checkpoints.^68^ The only viral protein expressed in all EBV-related malignancies is EBNA1, but the recognition of its character in carcinogenesis is limited. EBNA1 is essential for replicating and maintaining the EBV genome and can be used as an oncogene.^60^ A protein is involved in tumor suppression and regulates p53 activation of PML (Promyelocytic leukemia).^77^ If PML inhibits its activity, EBNA1 hinders p53-dependent p21 stimulation and apoptotic signaling, thereby improving cell survival in the case of DNA scarring.^71,77^ In addition, EBNA1 can prevent cell apoptosis by negatively regulating the expression of the oncogene Myc and intensifying the expression of antiapoptotic proteins such as Bcl2 and survivin.^68^

Furthermore, information about the induction of genomic instability is linked to the EBNA1.^71^ It also activates the production of reactive oxygen species (ROS), leading to chromosomal divergence. It is hypothesized that NOX2 is upregulated by EBNA, of which NADPH oxidase is the catalytic subunit, involved in the assemblage of subsequent chromosomal aberrations and ROS, telomere abnormalities, and DNA damage.^71^ EBNA2 is significant for preventing the apoptosis of transformed B cells and generating transformed B cells. EBNA2 cooperates with EBNALP and is responsible directly for initiating several viruses’ transcription (LMP1, LMP2A) and proteins of cells (CD21, CD23, MYC) that are important for B cell transformation and immortalization.^70^ Finally, the role of EBNA3 is to prevent the aggregation of CDK (cyclin-dependent kinase) inhibitors, deteriorate the protein RB (a tumor suppressor), balance the oncogene c-myc, and inhibit pro-apoptotic proteins.^78^ The cells which are EBV-infected express large amounts of RNA transcripts of the virus, called EBERs, which have exhibited to alter various processes at a cellular level, including growth factor production, cell proliferation, apoptosis, and signal transduction.^77^ EBER can alter miRNA expression to inhibit E-cadherin, leading to epithelial-mesenchymal transitions (EMT).^79^ EBERs assist chemoresistance by stimulating the STAT3/IL6 signaling pathway to down-regulate the cell cycle’s inhibitors of p21 and p27 expression.^80^ They also stimulate cell migration by activating pro-metastatic molecules pPAK1 and pFAK, inhibiting antimetastatic RhoGD1 fragments and KAI1.^77^ EBER protects cells from apoptosis mediated by IRF3 and NF-κB signaling and inhibits IFNα-mediated apoptosis resulting in the induction of growth-promoting cytokines such as IL6, IL9, IL10, and IGF1.^68^

### 2.5 Merkel Cell Polyomavirus (MCPyV)

MCC instances are uncommon, but their prevalence has risen in the previous two decades and is expected to rise even more. The disease rate for MCPyV in the European subcontinent of Scotland and France was recorded to be 0.13% per 100,000 individuals, and for Australia recorded at 1.6% per 100,000 individuals. Medical survey reports in the US recorded that the case rate was standing at 2500 cases yearly.^89^

MCC has a high fatality rate, with five-year overall survival rates of roughly 51% for patients with local disease at diagnosis and inadequate prognoses for those with more advanced stages. The MCPyV is a double-stranded nonenveloped DNA virus. It belongs to the family Polyomaviridae, Orthopolyomavirus, and different species of HPyV (human polyomavirus), such as BKPyV (polyomavirus BK) and JCPyV (JC polyomavirus). Its genome size is approximately 5.4 kb, divided into regions, i.e., early and late, by the specific regions known as the noncoding control region (NCCR). Three proteins are encoded by the early region: small T (sTag), large T antigen (LTag), and 57 kT (57 k Tag). Due to splicing alternatively, 78 amino acid sequences are shared by these antigens at their N-terminus, including the epitope of the B cell.^90^ This has also been observed in BKPyV;^91^ the expression of sT contributes to the production of antibodies against LTag, and this event should be noted. In MCC cells, insertions, deletions, and mutations can result in shortened LTag proteins within the Tag gene and Tag proteins of 57k. The late region encodes three capsid proteins (VP1, VP2, and VP3). In mammalian or insect cells, when expressed, the protein VP1 or (VP2 + VP1 protein) self-assembles within virus-like (VLPs) particles with a 45–55 nm diameter for serological tests.^92,93^ A very common viral skin infection, MCC (Merkel Cell Carcinoma), can cause unusual tumors because of the loss of immune surveillance. In addition to virus combination, LTag elimination, deletion of antigen T replication ability, and mutations of antigen T expression, MCC cell survival requires high antigen major capsid protein.^94^ MCC is generally seen as asymptomatic, with erythema, plaques, or violet masses, most commonly on smooth skin surfaces and sometimes telangiectasias. It is usually located in areas exposed to the sun, typically on the neck and head, followed by the trunk and limbs, but can affect any body part, including mucous membranes.^95^ The skin areas (lips, nipples, ruffles, and genital skin) mainly contain Merkel cells that are not particularly susceptible to MCC, and the source of MCC concern can be raised. In the case of MCPyV-positive, MCCs are not likely to have particular clinical characteristics; it is occasionally suggested that it is more often found in women and is confined to the limbs compared to the MCCs, which are MCPyV-negative.^96^ The related disease is present in the limbs at a rate of 66%, followed by disease of lymph nodes at 27%, and 7% of distant metastatic diseases are found.^97^ MCC can also be revealed by lymph nodes of unknown origin or distant metastasis, and there is no primary MCC in the skin or mucous membranes diagnosis.^98^

Recent studies on MCPyV have centered on the role of the virus’s encoded protein in tumorigenesis. Six out of eleven samples of MCC tumors were discovered to have MCPyV DNA integrated with them. The team quickly realized that viral DNA could be monoclonal integrated into the cell’s MCC genome by comparing the MCPyV DNA sequences in isolated metastatic tumors from different patients, suggesting that they occurred in cancer cells in the initial stage of the integration event before clonal expansion and tumor development.^99^ This early observation shows that the integration of the virus is a key occurrence in MCPyV-driven MCC oncogenesis in many other oncogenic viruses. Follow-up studies by various groups have affirmed that the genome of MCPyV is cloned and merged into the cancer genome in at least 80% of cases of MCC.^100−102^ Like other polyomaviruses, MCPyV LT has the conserved domains necessary to control the host cell cycle and replicate the viral genome. N-terminal LT comprises the DNAJ domain that binds to heat stroke protein, the motif protein binding the LxCxE (RB) of retinoblastoma tumor suppressor, and the conserved region 1 (CR1), which acts like the transformation of a cell area of the adenovirus E1A protein.^90^ The OBD (Ori binding domain) and the helicase/ATPase domain^103,104^ are located in the C-terminal region of LT and are essential for inducing viral DNA replication. Examining MCC tumors for LT transcripts expressed by the MCPyV incorporated into the genome.^90^ It was found that tumor-derived mutant LT truncation (LTt) sequence has mutations or premature stop codon deletions, which are the Ori Binding Domain C-terminal and domains of helicase required for dynamic viral replication, as to retain the binding motif of RB and the LT N items of other functional domains. Since the integrated virus is incompatible with viral DNA replication, it may inhibit tumor suppressor protein RB and control the host’s cell cycle. By contrast, similar LT mutational features were absent in MCPyV DNA obtained from a nontumor source.^90^ The sT and LT proteins are similar because they both include DNAJ and CR1 domains, but sT’s C-terminus is unique because it contains two protein phosphatase 2A (PP2A) binding motifs. MCPyV genome integration with MCC generally results in unaltered expression of the native sT antigen.^90,105^ Tumor regression in vivo was achieved with short hairpin RNA-mediated elimination of MCPyV sT/LT antigen in MCC cell lines.^105,106^ These studies demonstrate that MCC tumor cell growth in vitro and in xenograft models requires the expression of MCPyV LT and sT antigens. These innovative studies support the idea that MCPyV is harmful to the tumorigenic development of most MCC cancers.^105,106^ Two distinct MCPyV mutagenesis processes, namely, monoclonal coalescence of the virus’s DNA into the host genome and tumor-specific single MCPyV LT bridging, are required for initiating MCPyV-positive MCC tumor formation. The viral genome’s continuous replication state, exacerbated by the suppressive action of RB, may allow for the acquisition and accumulation of the essential genetic alterations by newly forming tumor cells as they increase, allowing them to progress to the MCC stage. The inactivation of RB is a crucial step in the occurrence of MCC tumors. Although most negative-MCPyV MCCs transfer gene mutations/deletions deactivate the cell’s RB1 gene,^107−109^ positive MCPyV MCCs usually encode the wild-type gene RB1.^65,108^ However, all familiar MCC-derived LTt mutant truncation retains the RB motif binding,^90^ allowing binding of them to the factors of tumor suppressor with higher affinity.^110,111^ These examinations specify that the interaction between the RB and MCPyV may be essential in promoting the malignant progression of positive-MCPyV tumors. Integration of proviruses like MCPyV into oncogenic MCC cells that generate LT truncation mutants usually results in losing the DDR activation domain.^90^ C-terminal LT truncation mutant is more potent in encouraging cell growth than full-length LT protein.^112^ This suggests that virus-induced host DDR may be a barrier to malignant development. Therefore, in MCPyV-driven tumorigenic development, releasing the tumor brake of a suppressor can remove the innate DDR-inducing and growth-inhibiting activity of the domain C-terminal of MCPyV LT, allowing tumor growth.^113^

It is worth mentioning here that MCPyV diverges significantly from other polyomaviruses. MCPyV sT, in contrast to sT from other polyomaviruses, plays an active role in the virus’s ability to modify host cells. sT is more commonly found than LT antigen in MCPyV-positive MCC tumors and is present in most MCC tumors.^114^ In MCC cells, overexpression of LTt does not fully restore growth inhibition when MCPyV LTt and sT are removed.^106^ These findings suggest a critical function for sT in MCPyV-related tumor progression. A sustained expansion of MCC cell lines that test positive for MCPyV requires the expression of sT. The expression of sT alone is sufficient to convert rodent fibroblasts in the non-anchored lesion development assay. Hyperphosphorylation of eukaryotic initiation factor 4E binding protein 1, a critical downstream target of the PI3K/mTOR/AKT signaling cascade, leads to the overactivated cap and, hence, sT’s transformation potential. mRNA is required for cell translation.^114^ Inhibition of PP2A’s ability to stimulate cell proliferation is one-way. The other way can affect the AKT signaling pathway.^115^ Despite MCPyV sT’s ability to bind PP2A, it does not appear that PP2A inhibition is necessary for transformation activity.^114,116^ Traditionally, Merkel cells tend to produce MCC because the neurosecretory granules in the tumors indicate the origin of the neural crest^117^ and also express the unique Merkel cell marker, cytokeratin 20.^117^ It is suggested that tumors of MCC may originate from the lineage of B cells because they are willing to express pro/pre-B markers of cells.^118^ Since most dermal MCC cancers start in skin fibroblasts, the discovery that MCPyV may infect these cells is consistent with this.^119^ There is evidence that MCPyV infection of skin fibroblasts can drive their transformation into other cell types (such as B cells, skin cells, and Merkel cells) under as-yet-unknown circumstances. New evidence suggests that skin fibroblasts may be able to undergo a self-renewal process and transform into MCC cells by expressing the MCPyV LTt. MCC cells are characterized by a neuroendocrine-like development pattern.^120^ On the other hand, Merkel cell precursor virus (MCPyV) actively replicates in dermal fibroblasts and may accidentally reach the precursor cells of neighboring Merkel cells.^121^ This uncontrolled propagation environment can promote cancer when these cells differentiate into Merkel cells and the replication of defective MCPyV genome integrates into the host cell genome. These two ideas, MCC arising from dermal fibroblasts and MCPyV infection via nonproductive transient Merkel cells, will need to be tested once an in vivo infection model of MCPyV is available.^121^

### 2.6 Hepatitis C Virus (HCV)

Liver cancer is the sixth most prevalent cancer worldwide and the fourth main cause of cancer death. A record population of more than 150 million people were diagnosed with chronic HCV in 2005. The caseload of HCV ranges from 4 to 16% in Asian and African inhabitants, with a ≥ 20-year band gap population covering around 4% of the total caseload. The strait of Taiwan is reported to have the highest incidence rate of HCV cases. The developed nations of North America, the western and northern subcontinents of Europe, and Australia are geographic zones with a low frequency of HCV infection. Some developed regions with relatively minimal HCV rates include Germany recording 0.6%; Canada recording 0.8%; Australia and France recording 1.1% seropositivity levels. The HCV seropositivity levels in the US were 1.8%, Japan with around 1–3% seropositivity levels, and Italy recorded 2.2% seropositivity levels.^122,123^ Hepatocellular carcinoma (HCC) is the most common liver cancer, accounting for 70–80% of cases. Chronic infection with the hepatitis C virus (HCV) has been identified as a key driver of HCC.^124^ According to mounting evidence, hepatocarcinogenesis is linked not only to inflammation and consequent fibrosis but also to HCV. It has been demonstrated in experiments employing transgenic mice and cell-culture models in which HCV proteins are produced.^125^ The Hepatitis C virus is an enveloped compact RNA virus that belongs to the family Flaviviridae and the Hepacivirus genus. HCV RNA genome is single-stranded with positive polarity, surrounded by a core protein and a bilayer of lipids that consists of two viral glycoproteins (E1 and E2) to form virus particles.^126^ Although there are differences in nucleotide sequence between the genetic constitution, all currently identified Hepatitis C virus genotypes are pathogenic and hepatotropic.^127^

Hepatitis C spreads through the blood contact of a diseased person. Nowadays, most people get infected with HCV by sharing syringes or other equipment to prepare and inject drugs. For a few people, hepatitis C is a limited condition illness. Still, for more individuals who get infected with the hepatitis C virus, it can turn into a deep-rooted chronic infection. Hepatitis C, which is chronic, can cause consequential and even lethal health problems such as liver cancer and liver cirrhosis. People with chronic hepatitis C generally have no symptoms or discomfort. When the appearance of symptoms takes place, those are usually signs of the advanced stages of liver infection. There is no vaccination available against hepatitis C. The best method to block hepatitis C is to avoid behavior that can spread the disease, especially by injecting drugs. Diagnosis for hepatitis C becomes crucial because treatment can cure most patients with the infection within 8 to 12 weeks.^128^

The life cycle of HCV initiates with the binding of virus particles to distinct receptors on liver cells.^129^ So far, class B class I HDL receptor scavenger receptor, CD81 four-span membrane protein, claudin1 tight binding protein, and occludin are known cell receptors that can initiate the binding phase infection of HCV. It is suggested that the virus is internalized after it is bound to its receptor complex and the nucleocapsid is released into the cytoplasm. Then, the virus evolves to release its genomic RNA, which is used for polyprotein translation and replication in the cytoplasm. Replication of the hepatitis C virus occurs in a ″replication complex″ containing nonstructural proteins of the virus and cellular proteins.^130^ Also, the HCV replication is catalyzed by the NS5B protein. However, there are essential viral proteins that are found to be nonstructural. The helicase/NTPase domain of protein NS3 has a variety of significant functions for virus replication, including the RNA-activated action of NTPase, RNA binding, and unwinding of RNA regions with a considerable secondary structure.

The formation of a replication complex is initiated by NS4B, which supports the replication of HCV. The protein NS5A also plays a crucial regulatory role in virus replication. New antiviral drugs that are direct-acting (DAA) are now on the market, particularly designed to hinder RNA NS5B-dependent RNA polymerase enzyme. Several newer DAAs (such as protein NS5A inhibitors) have also shown promising results in clinical studies.^131,132^ In HCV replication, many cytokines are involved, such as cyclophilin A, which is necessary for the replication of HCV by interacting with NS5B and NS5A, and microRNA122, which assists by binding to the 5′ untranslated domain (5′UTR) of HCV, and then the genome replicates. Therefore, factors of the host can also become potent targets for the therapy of anti-HCV. According to a recent study, at least two targeting agents of the host (HT) have clinically reached the stage of development, including microRNA122 antagonists and cyclophilin A inhibitors.^131,133,134^

**Figure 2:**
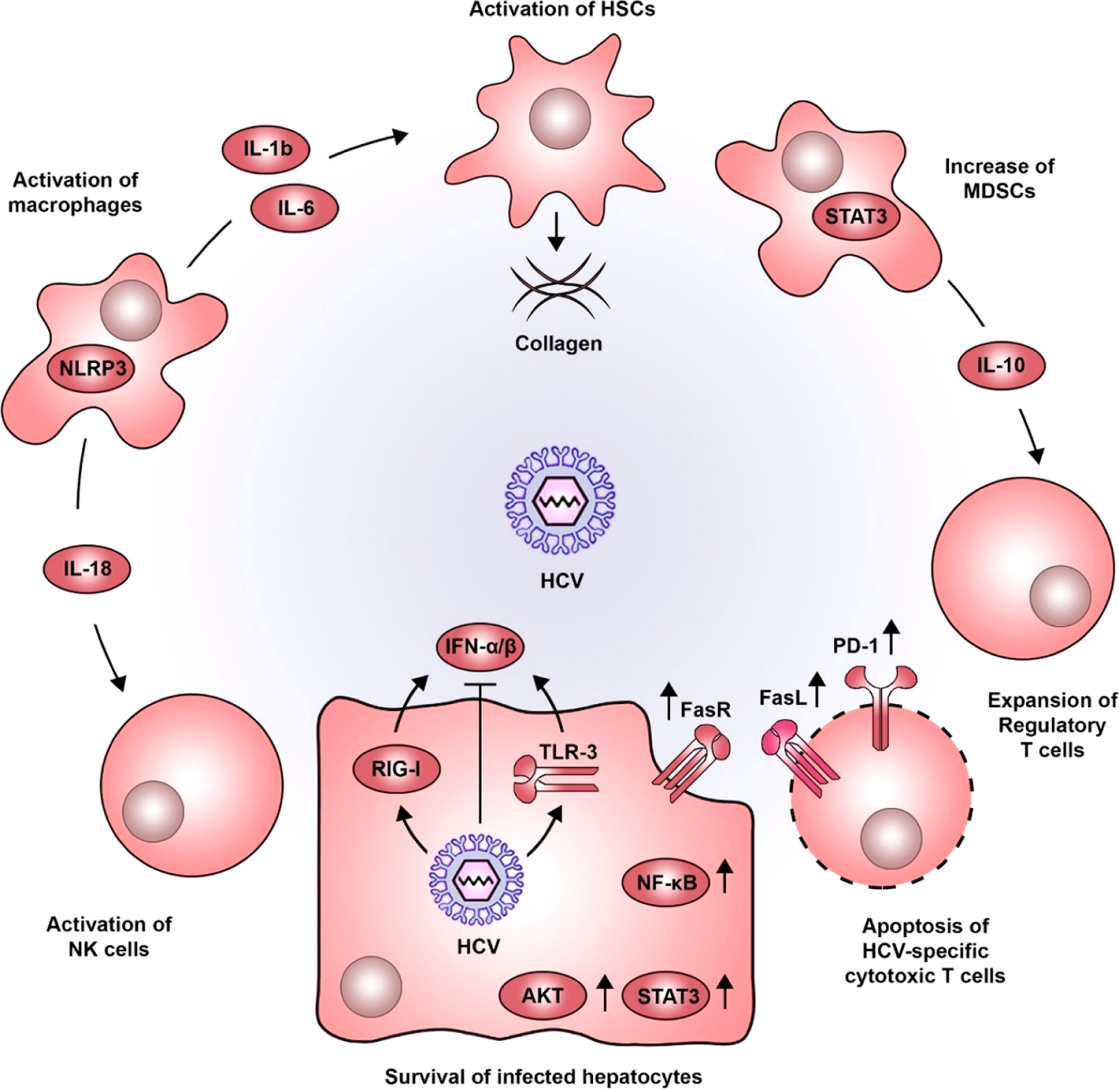
Pro-oncogenic inflammatory microenvironment induced by HCV. HCV infection in hepatocytes is detected by viral sensors, such as RIG-I and TLR3, leading to the production of type I IFNs. As with most viruses, HCV has developed various strategies to dampen this antiviral response. The persistent inflammatory environment in the liver, combined with the action of viral proteins, establishes a sustained activation of signaling pathways associated with cell survival (e.g., STAT3, AKT, NF-κB, and FasR). Sensing HCV-infected hepatocytes by macrophages triggers NLRP3 inflammasomes, inducing the secretion of IL-18, which activates NK cells. Moreover, IL-1b and IL-6 produced by macrophages favor the activation of HSCs which are central components in the progressive deposition of collagen associated with liver cirrhosis. STAT3 also plays a role in developing MDSCs, which produce IL-10 and favor the expansion of regulatory T cells. This altered immune response is further accentuated by the increased expression of PD-1 and FasL, impairing cytotoxic T lymphocyte function and inducing their apoptosis (adapted with permission under a creative commons (CC-BY 4.0) from ref (124)). Copyright [2020] [MDPI].

The mechanism involved is appropriate low-density lipoprotein synthesis/secretion in the production of infectious fragments of HCV. The Hepatitis C virus uses this lipoprotein biosynthetic pathway to generate mature particles of the virus and exports them.^132,135^ Growth factors such as EGF (epidermal growth factor), FGF (fibroblast growth factor), HGF (hepatocyte growth factor), and IGF (insulin growth factor) trigger downstream signal transduction by binding to their specific tyrosine kinase receptors.^136^ The cascade of occurrence after the EGFR (epidermal growth factor receptor) is the signal transduction pathway studied most widely.^137−139^ ErbB1 and the other three analogous members of the family EGFR (ErbB2, ErbB3, ErbB4) modulate cell differentiation, proliferation, and migration under normal physiological state.^138^ EGFR is essential for epithelial development, and other family members are essential in breast, heart, and nervous system development and diseases.^137,140,141^ The EGFR also plays a key role in developing embryo and stem cell regeneration into the skin, liver, and intestines.^142^ In addition, the EGFR has also attracted much focus as a determinant risk of cancer and progression.^143,144^ Viruses have established complex methods to manipulate the function of EGFR (i.e., disrupt EGFR expression, activity, or recycling).^145^ The host factor for HCV is EGFR, by regulating complex coreceptor assembly to enter hepatocytes,^146,147^ viral internalization,^148^ and membrane fusion.^146^ In addition, the signaling pathway EGFR maintains phosphorylation of the signal transducer and activator of transcription 3 (STAT3) by hindering negative feedback regulators (i.e., the cytokine suppressor 3 signaling, SOCS3), thus regulating the effect.

(146) HCV is important for controlling the signaling of EGFR. Indeed, the hepatitis C virus involves the signal transduction of EGFR and actively induces activation of this pathway during HCV binding and infection^149,150^ and prolongs EGFR signal transduction by interrupting the degradation of EGFR through NS5A, as described by its ectopic expression.^151^ This can result in an increased threat of HCC in infected patients because continuous signaling of EGFR is a leading factor in hepatic disease.^143^ The transforming growth factor-β (TGFβ) of cytokine dimers of the superfamily with a pleiotropic and conserved structure. Under physiological conditions, TGFβ acts as an effective growth inhibitor for various cell types;^152,153^ and promotes epithelial cell apoptosis^154^ (Figure 2). Thus, damaged TGFβ can lead to excessive cell proliferation and cancer.^155^

Furthermore, these cytokines stimulate the expression of extracellular matrix components and build up fibrosis in different tissues in vivo.^154^ In the case of the liver, TGFβ appears to contribute to all phases of disease development, from early damage to inflammation and fibrosis, through cirrhosis and HCC.^156,157^ In early cancer development, TGFβ may act as a tumor suppressor. Still, once tumor cells become resistant to its suppressive properties, it will promote tumor progression, migration, and invasion in advanced HCC.^156,158^

The key effector of intracellular signal transduction is Smad4. Like TGFβ, it has duplex tumor suppressor and HCC promoter roles.^159^ In the nucleus, the transcription of target genes is regulated by the SMAD complex induced by TGFβ and the necessary cofactors involved in transcription. Genetic characteristics are specifically induced by the SMAD complex through the typical TGFβ signaling pathway,^160^ causing growth arrest and pro-apoptotic indication in the early stage. Later, generative and antiapoptotic feedback gain advantages through crosstalk with growth signals. This nonclassical TGFβ pathway includes regulating EGFR by small GTPase signaling pathways, MAPK (mitochondrial activated protein kinase), PI3K (phosphoinositide 3-kinase)/Akt, Ras, and Rho-like.^161,162^ TGFβ can induce EMT in primary hepatocytes of humans, which is a procedure that stimulates metastasis and cell invasion.^163^ Epithelial cells lose their phenotypic attributes and gain invasiveness during TMS, becoming cells of mesenchymal. EMT is necessary physiologically for embryonic development. However, there is an escalating affirmation that it also plays a role in pathological conditions and may contribute to the development of metastatic oncogenesis.^164^ A captivating hypothesis suggests that chronic infection causes HCV selection for protumorigenic mutations in the liver, which strongly interferes with TGFβ signaling. Core variants from HCC support the isolation of HCV. Compared with hepatitis C virus core forms isolated from adjacent tumor tissues, HCV core variants can better resist TGFβ-mediated antiproliferation effects and promote cell transformation.^165^ Its association with SMAD and a core expression of HCV on the surface of infected liver cells activate endoglin’s expression (CD105). As a constituent of the TGFβ complex receptor, abundant endoglin triggers fiber production and promotes the growth of tumors and metastasis.^166^ Endoglin induces the inhibitory function of DNA binding 1 (ID1) by stimulating ALK1/SMAD1/5 signaling, which plays a role in proliferation and antiapoptosis. It is a primary regulator of CSC maturation.^167^ Infection of HCV or ectopic articulation of the viral core elevates the expression of ID1-associated CSC, proliferation, and survival markers (i.e., NOTCH1, BCL2, HES1, CyclinD1, NANOG, and SOX2 proteins).^166^

Furthermore, endoglin is a marker of angiogenesis in patients with HCC.^168^ The Hedgehog (Hh) pathway coordinates key morphogenesis operations, including propagation, survival, migration, and differentiation.^169^ Hedgehog ligands are necessary during embryogenesis and morphogenesis and for maintaining stem cell homeostasis in adulthood. Importantly, the Hh pathway plays an essential role in hepatic repair and regeneration in adults^170^ and is associated with many types of liver malignancy, such as cancer of the gallbladder, cholangiocarcinoma,^171−173^ hepatoblastoma,^172^ and HCC^174^ accumulation of markers of liver damage (i.e., PDGF (platelet-derived growth factor), TGFβ, and EGF) may lead to the production of Hh ligands.^175,176^ In patients with hepatitis, the Hh pathway was found to be generated^177^ and may reflect the damage of tissue and regeneration of the liver during chronic infection. Strikingly, the cell’s permissibility for replication of HCV appears to be pragmatically correlated with the action of the Hh pathway,^178^ indicating that regeneration of the liver and an environment of profibrosis may stimulate the infection of HCV. The recognition of other key regulators of liver regeneration initiated by HCV infection supports this, which includes signaling of EGFR^146,149,150^ and IL6/STAT3.^179^ Furthermore, the Hh activity promotes crosstalk between EMT and TGFβ and Wnt signals,^180^ which once again highlights the correlation between the induction of EMT and hepatitis C virus and its repercussions for HCV-related hepatic pathogenesis and the progression of HCC. The summarized version of the viral strains, their genomic material, and their oncogenic property has been highlighted in Table 1.

**Table 1:** List of Viruses and Their Related Malignancies

S. No.	Viruses	Genome	Types of Cancer	References
1.	Merkel cell polyomavirus (MCPyV)	Double-stranded DNA Polyomaviridae	Merkel cell carcinoma	(25)
2.	Hepatitis B virus (HBV)	Partially double-stranded DNA Hepadnaviridae	Hepatocellular carcinoma	(81)
3.	Human Papilloma virus (HPV)	Double-stranded DNA Papillomaviridae	Cervical, neck, head, and anogenital tract carcinoma	(82,83)
4.	Kaposi sarcoma herpesvirus (KSHV/ HHV-8)	Double-stranded DNA Herpesviridae	Primary effusion lymphoma, Kaposi sarcoma, multicentric Castleman disease	(84)
5.	Epstein–Barr virus (EBV/HHV-4)	Double-stranded DNA Herpesviridae	Burkitt lymphoma, Hodgkin lymphoma, nasopharyngeal carcinoma	(85)
6.	Hepatitis C virus (HCV)	Single-stranded RNA Flaviviridae	Hepatocellular carcinoma, Lymphomas	(86)
7.	Human T-cell leukemia virus- (HTLV-1)	Single-stranded RNA Retroviridae	Adult T-cell leukemia and myelopathy/tropical spastic paraparesis	(87)
8.	Human immunodeficiency virus (HIV)	Double-stranded RNA Retroviridae	Elevates the immunosuppression-mediated malignancies by other oncogenic viruses	(88)

### 2.7 Human T-cell Lymphotropic Virus (HTLV-I)

The geographic distribution of the virus has been defined. However, some puzzles persist, such as the high prevalence in southwestern Japan but the low prevalence in neighboring regions of Korea, China, and eastern Russia, and seemingly isolated pockets of infection in Iran. The seropositivity levels for HTLV1 in parts of Europe and North America were reported to be low. In the United States and Canada, seropositivity levels were recorded to be 0.01–0.03%; in Norway, 0.002% was recorded, and in Greece, 0.0056% was recorded. Higher seropositivity levels of about 10% were recorded in the Southwestern Japan flank among pregnant females and blood donor individuals. Following Japan, Caribbean countries such as Jamaica and Trinidad present around 6% seropositivity levels for HTLV1. In the Sub-Saharan Africa basin like Benin, Cameroon seropositivity levels of around 5% are reported. In parts of the Middle East like Iran and Melanesia, HTLV1 seropositivity levels were reported at ≤ 5%. In South American countries, namely Argentina, Brazil, Columbia and Peru, seropositivity for HTLV1 was recorded at around 2% specific to samples of pregnant females and native individuals.^181,182^ HTLV-I is the only human pathogen of this oncogenic virus subfamily, an encapsulated single-stranded RNA virus belonging to the Retroviridae family, including HTLVII, bovine leukemia virus (BLV), simian T-cell leukemia virus (STLV), HTLV-III and HTLVIV.^183^ It contains a diploid genome comprising two 9032 base pair long positive-strand RNAs. The gag, pol, and env genes are flanked by two long terminal repeats (LTR) sequences comparable to those in other retroviruses. However, between env and 3LTR, HTLV-I possesses a unique 1.6 kb region known as pX.^184^ This region encodes many regulatory proteins: p40tax (Tax), p27rex, p21rex, p12, p13, and p30. The basic leucine zipper factor HTLV-I (HBZ) is encoded by the negative (complementary) strand of the pX region.^185^ Among them, Tax and HBZ are related to the pathogenesis of the virus. (See ″Pathogenesis″ below). Cell entry and replication compared with HIV infection, the level of viremia in the HTLV-I infection is extremely low. The new infection results from the spread of infected lymphocytes rather than cell-free virus particles. HTLV-I is CD4 T cell-specific, although virus particles penetrate CD4 T cells more efficiently via direct cell-to-cell contact across viral synapses^186^ than plasma-free virus particles^187^ observed that cell-free HTLVI could infect dendritic cells (through heparan sulfate proteoglycan and neuropilin 1), which can then expand to CD4 T cells.^188^ The glucose transporter GLUT1 has also been discovered as a receptor for the envelope glycoprotein HTLV-I.^189,190^ GLUT1-deficient cells, on the other hand, can infect HTLV-I.^191^ CD4-positive T cells infected with HTLV-I can produce CCL22, a CCR4 ligand. CCL22 attracts CD4 cells expressing CCR4, leading to preferential transmission of HTLV-I to CCR4-positive and CD4-positive T cells.^192^

Although free viral particles have been found to infect dendritic cells, HTLV1 infection occurs predominantly and most efficiently through disseminating infected lymphocytes.^193^ CCL22 (a CCR4 ligand) is produced by infected CD4+ cells, which bind to CCR4 on CCR4+ CD4 cells, forming what is known as ″viral synapses.″ In study,^194^ HTLV1 is more effectively spread among the CD4+ CCR4+ (TC) T cell population. Although HTLV1 and HIV may both infect TC, there are significant variations in their virology and final pathogenesis (HIV). One such distinction is this distinctive pattern of cell-to-cell communication. When compared to the high viral load of HIV, the viremia associated with HTLV1 is rather mild. High genetic stability is a second distinguishing feature of HTLV1, which is ensured by the virus’s reproduction method.^195^ The DNA product of the HTLV1 genome is introduced into the host genome once the virus enters a cell and undergoes reverse transcription. The virus has two options for further replication: infectious replication, in which case reexpression of the integrated provirus will generate new intracellular viral particles, or mitotic replication, in which case the integrated provirus will reproduce itself. Viral replication is inextricably related to the reproduction of the host cell, unlike the case with independent viral DNA polymerases.^196^ This results in a stable gene product (not the HIV gene product) that circumvent immunological escape by maintaining a low viral replication rate and high transcription fidelity.^197^ HTLV1 can regulate its transcription, so the transient expression can help evade host immune control gene products.^198^ Two regulatory proteins facilitate this: the Tax mentioned above (activation of transcription) and Rex (repression of transcription).^199^ Integrating the provirus and the translation of viral products is related to cell proliferation and an increased survival rate, which confers protection against the virus. Importantly, unlike HIV, HTLV1 infection does not cause cell death. On the contrary, TC can escape apoptosis and is easily transformed.^200^

Adult T-cell leukemia/lymphoma (ATL) is etiologically linked to HTLV1.^182^ HTLV1 has more intricate genetic makeup and regulation than other leukemia viruses. Besides the structural genes encoding functional viral proteins (gag, pro, pol, and env), the HTLV1 genome also includes genes encoding the nonstructural proteins Tax and HBZ, which are important in controlling the viral gene.^201^ Although large advancement has been done in comprehending the complicated mechanisms of ATL due to HTLV1, research has nevertheless had to make clear the role and control of viral gene products and their interactions with each other, in addition to the interplay with cytokines.^201^ Previous research showed that Tax1 is mainly located in the nucleus, especially accumulating in the mottled structure of the nucleus. Recently, it has been mentioned that Tax1 is likewise positioned within the cytoplasm,^201,202^ even though the mechanism that regulates the subcellular localization of Tax1 has not at this time been explained. The N-terminal domain of Tax1 consists of a binding vicinity with CREB72, which is vital for its interplay with proteins required in cell cycle advancement, transcription, and management of cell signaling.^203^ Besides, Tax1 interacts with cytokines, which include the reaction of cAMP-response element binding protein (CREB) and the transcriptional coactivator CBP/p300.^204^ As a viral oncoprotein, Tax1 performs a key function in tumorigenesis. It involves ATL by regulating many intracellular signaling pathways, including the IκB kinase (IKK)/NF-κB^205^ signaling pathway, DNA repair pathway, and innate immune signaling pathway. Pathogenesis includes RIGI/MDA5-dependent and TLR-independent pathways, TRIF-dependent TLR pathways, and the lately explored cGASSTING pathway.^206,207^ The typical characteristics of tumor cells are genetic and phenotypic instability, called mutant phenotype.^208^ Genomic harm can arise because of internal (metabolic) and outside factors (genotoxic stress) and DNA replication mistakes.^209^ Generally, those errors are corrected immediately through many cellular restore mechanisms.^208^ If those repair pathways are not carefully coordinated, genomic lesions can form mutations in cellular division and DNA replication, prompting genome instability.^210^ It is widely believed that increased mutations in the cellular genome and the suppression of the Tax1-mediated DNA repair pathway are two hallmarks of HTLV1-transformed cells.^209^ These alterations indicate the protein’s ability to inhibit DNA repair pathways such as base excision repair, nucleotide excision repair, mismatch repair, non-homologous end joining, and homology-directed repair (homologous recombination).

For the NER pathway, specifically, it has been shown that Tax1 can hinder the pathway via transactivation of PCNA (proliferating cell nuclear antigen). PCNA is essential for DNA replication and repair as it acts as a cofactor for DNA polymerase.^211^ Additionally, Tax1 can impede tumor suppressor action by inactivating p53.^212^ When it comes to NER, Tax1 acts in a dual dose-dependent fashion. Researchers have identified a novel open reading frame (ORF) on the HTLV1—a negative strand that encodes a basic leucine zipper factor termed HBZ.^185^ Genome integrity, cell proliferation, apoptosis, autophagy, and immune evasion are only some of the cellular processes that HBZ regulates.^213^ By building heterodimers with host factors such as CCAAT/alpha-enhancer binding protein (C/EBPa) and activating transcription factor 3 (ATF3), HBZ controls cell proliferation (ATF3). In many cases, C/EBPa suppresses cancer cell growth.^214^ By interacting with C/EBPa and decreasing its DNA binding ability, HBZ can counteract the growth-inhibitory effects of C/EBPa and stimulate cell proliferation. There are two sides to the tumorigenic action of the HBZ-binding protein ATF3. ATF3 is a tumor suppressor because it promotes p53 signaling.^215^ However, it stimulates the growth of cancer cells.^185,215^ ATF3′s p53 enhancer function is negatively affected by HBZ, which is detrimental to ATL development, although ATF3′s function in cell proliferation is unaffected by HBZ in ATL cells.^215^ Two investigations have demonstrated that HBZ promotes ATL cell proliferation via autocrine and paracrine mechanisms.^216^ ATL cell proliferation and migration are both improved by HBZ because of its effect on the production of the noncanonical Wnt ligand Wnt5a, whereas the canonical Wnt pathway is inhibited. Furthermore, HBZ promotes ATL cell proliferation by elevating the expression of brain-derived neurotropic factor (BDNF) and its tropomyosin receptor kinase B (TrkB).^216^ Double-stranded breaks (DSB) induced by HBZ are based on various microRNAs (miRNAs) that can induce HBZ, such as miR17 and miR21.^217^ miR17 and miR21 target and inhibit the expression of OBFC2A. OBFC2A is a gene encoding hSSB2, and hSSB2 is a single-stranded DNA-binding protein that prevents genome instability.^217,218^ Therefore, HBZ destroys the integrity of the host genome through the HBZ microRNA OBFC2A cascade.^218^ Studies have shown that among the HTLV1 viral proteins, HBZ has the lowest immunogenicity because anti-HBZ antibodies are almost undetectable in individuals infected with HTLV1.^219^ A recent study showed that the weak binding strength of HBZ epitopes to CTL and low expression of the HBZ protein could greatly hamper the host’s ability to successfully initiate an anti-HBZ CTL response. It has been suggested that the low immunogenicity of HBZ may help infected cells to evade immune surveillance and contribute to HTLV1-mediated tumorigenesis.^220^

### 2.8 HIV (Human Immunodeficiency Virus)

The human immunodeficiency virus (HIV) isolates are currently categorized into two types: HIV type 1 (HIV1) and HIV type 2 (HIV2). HIV1 is the most common cause of AIDS worldwide, while HIV2 is only found in portions of West and Central Africa. HIV belongs to the Lentivirus genus of the Retroviridae family and is genetically connected to it. Lentiviral infection usually has a chronic course, long clinical incubation, persistent virus reproduction, and central nervous system involvement.^221^ The retroviral genome comprises of two identical copies of single-stranded RNA molecules, and structural genes gag, pol, and env. Although the basic structure (three structural genes, gag, pol, and env) is the same in all retroviruses, the genome structures of HIV1 and HIV2 viruses differ. The HIV1 and HIV2 genomes contain complicated combinations of other regulatory/helper genes and these three genes. Even though central nervous system disorders are more common in HIV2 infection,^222^ AIDS can be caused by any of these two viruses. In addition, HIV2 seems less virulent than HIV1, and the infection process takes longer to develop into AIDS.^223^ The HIV particle structure of HIV1 and HIV2 is comparable to those of other retroviruses, as seen in Figure 4.

The gag gene produces matrix proteins (p24, p7, and p6) as well as core structural proteins (p17). The viral envelope glycoproteins gp120 and gp41, which recognize cell surface receptors, are encoded by the env gene. The pol gene encodes enzymes required for viral replication. They are reverse transcriptase, which transforms viral RNA into DNA; integrase, which integrates viral DNA into host chromosomal DNA (protovirus); and virus protease, which breaks the virus’s RNA protease. The predecessors of the Gag and Pol proteins are broken down into their constituents. HIV has a particle diameter of 100 nm and is enveloped by a lipoprotein-rich membrane. A glycoprotein heterodimer complex made up of an outer surface of gp120, and a transmembrane glycoprotein trimer of gp41 is found on each viral particle membrane. Because the interaction of gp120 and gp41 is not covalent, gp120 can be lost spontaneously in the immediate environment and detected in HIV-infected patients’ serum and lymphatic tissue. The virus can also draw from diverse host cell membrane proteins, such as HLA class I and class II proteins, or adhesion proteins, such as ICAM1, during the budding process from infected cells and aids in the attachment of additional target cells. Within the viral lipoprotein membrane, matrix protein (p17) is attached. A capsid of polymers of the core antigen is included in the viral membrane and matrix protein (p24). Two copies of HIV RNA are coupled with a nucleoprotein, reverse transcriptase, integrase, and protease in the capsid.^224^

Another feature of HIV is the presence of helper/regulatory genes that regulate the virus’s replication.^225^ The tat gene, for example, encodes a protein (Tat) produced shortly after infection and increases HIV gene expression. The rev gene encodes the Rev protein, which ensures that it is exported from the nucleus to the cytoplasm as properly processed messenger and genomic RNA. Other HIV helper proteins’ roles are not well understood. Vpr protein is thought to be involved in cell cycle arrest. This protein also enables the reverse transcription of DNA into the nucleus of nondividing cells (such as macrophages), a function that Vpx in HIV2 does. The vif gene encodes a small protein (Vif) that can boost the infectivity of progeny virus particles. Vpu is a protein that is important for properly releasing viral particles, and the vif gene encodes a small protein (Vif) that can enhance the infectivity of progeny virus particles. Finally, the Nef protein has a variety of activities, including cell signaling and down-regulation of the CD4 receptor on the cell surface, which allows the virus to bud late in the replication cycle.^225^

**Figure 3:**
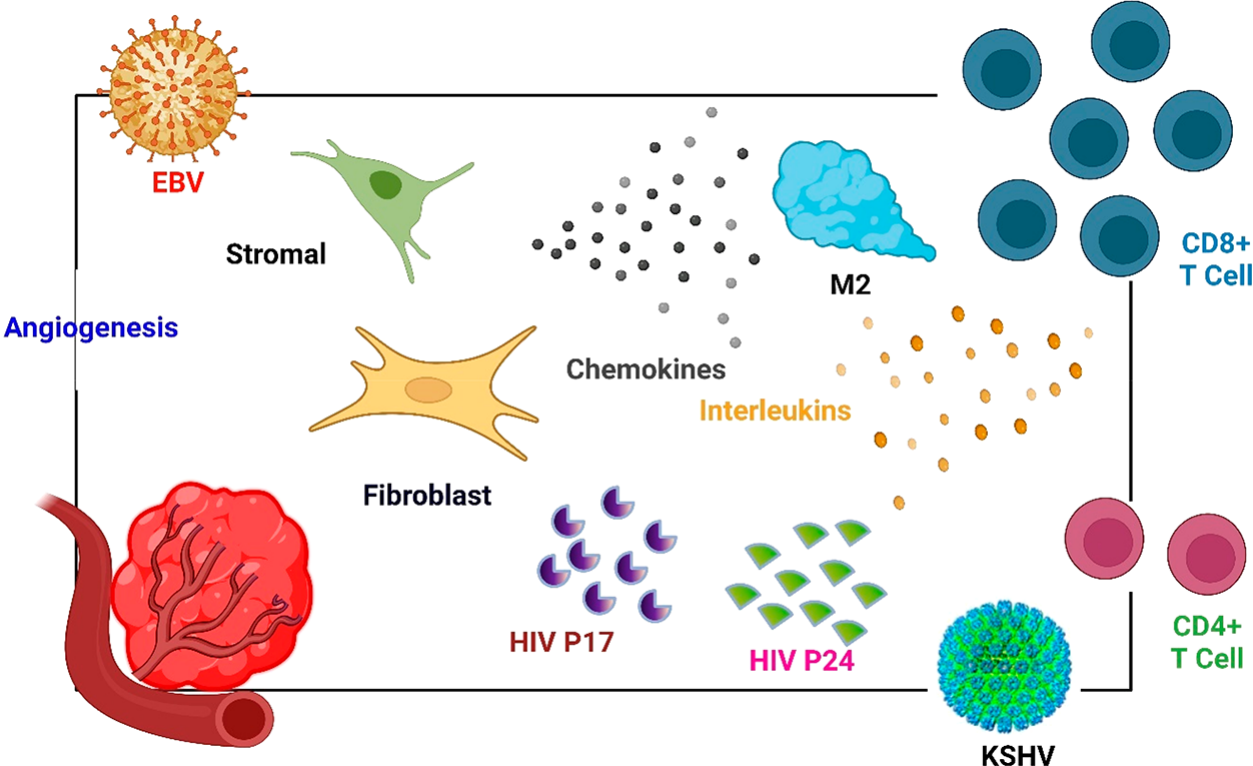
Schematic representation of the lymphoma microenvironment including immune components (CD4 and CDT lymphocytes, macrophages, cytokines, and chemokines) and nonimmune components (fibroblasts, stromal cells, and blood vessels). This figure includes viruses and their products influencing the microenvironment (Created in Biorender).

Even though there is a definite association between HIV1 infection and the development of some malignancies, it is unclear whether HIV1 acts as a carcinogen directly. Viral-induced carcinogenesis appears to depend on several conditions in the setting of HIV-1 infection, the most common of which include coinfected virus cooperation and anomalies in the immunological and nonimmune microenvironments. In this regard, we will discuss the mechanism of HIV1-associated tumorigenesis, concentrating on virus products’ direct ability to promote tumorigenesis in cells and the indirect cause of inducing cancer. As a result, significant immunological diseases develop.^226^ The importance of viral cofactor cooperation in the malignant transformation of HIV1-infected persons is stressed. High immunodeficiency and chronic immune activation/inflammatory state will enhance vulnerability to infection and multiple replication pathways.^227^ Since the direct and transient depletion of T cells and the microenvironmental changes that define pathogenic HIV1 infection led to the failure of immune surveillance, it makes sense that HIV1 replication derivates may also result in tumorigenesis.^228^ The contribution of HIV1-induced molecular changes in the generation of tumor transformation independent of immune system abnormalities have been proven, mostly using in vitro cell line models. The direct cooperative involvement of HIV1 in mediating carcinogenesis has been established in the context of KS. The current evidence suggests that the chronic immunological inflammatory state associated with HIV1 illness contributes to B cell activation and, eventually, lymphoma; HIV1 replication products may also contribute directly to lymphoma induction. Through its association with DCSIGN, the HIV1 envelope protein gp120 can directly stimulate B cells, causing class switching and recombination of immunoglobulin genes, interleukin release, and AID-induced activation (Figure 3).^229^ The interaction of HIV1 envelope interacts with a range of cellular proteins with different functions, such as adhesion molecules, MHC components, macrophages, and B and T cell surface proteins. The chemicals aid in stimulating B cells and developing lymphoma.

Many immune cell components, such as activated T cells, B cells, and macrophages, are found in the tumor microenvironment. The role of the interaction of HIV1 with the microenvironment has been increasingly highlighted in recent years. As previously stated, HIV-1 infection can severely deplete cytokine and chemokine levels while encouraging antiviral immunity formation. However, these mediators can harm the host by restricting the establishment of a strong immune response to infection and influencing cancer development directly or indirectly.^230−232^ Increased IL6, IL10, CXCL13, and TNFα are linked to an increased risk for NHL in HIV1-infected persons.^233^ The activation of pro-inflammatory mediators by HIV1 infection may alter the development of other immune regulatory factors, cell proliferation, apoptosis sensitivity, and other microenvironmental physiological processes.^227^ As previously discussed, the HIV1-dependent transition of T helper (h) 1 cell subsets to Th2 CD4+ is thought to be a crucial stage in immunological dysregulation. Increased Th2 differentiation could be a precursor to AIDS-related NHL and a factor in Th17 overexpression.^234^

Furthermore, Th2 or Th17 cytokines induce AID expression in the pathogenesis of AIDSNHL.^233^ In HIV-1 infected patients, cytokine/chemokine system dysregulation may also induce neovascularization. In addition to Tat’s role in boosting capillary development,^235^ HIV1 matrix protein p17 has been found to stimulate angiogenesis through chemokine receptor linking through various pathways.^236^ In more detail, p17 is released from HIV1-infected cells and binds to CXCR1, after which CXCR1 pushes human monocytes into the microenvironment like IL8 chemokines, resulting in long-term inflammation.^237^ Increased levels of IL6 and TNF in HIV1-infected individuals’ plasma can cause the production of COX2 and PGE2, which has been linked to the development of AIDS-related cervical cancer,^238^ and this impact could be linked to angiogenesis.^238^

Further research is needed to determine the specific role of HIV1-driven angiogenesis in cancer formation and progression. Finally, the nonimmune microenvironment may play a role in the occurrence and progression of AIDS-related malignancies, possibly through virus-cooperative mechanisms. Endothelial cells, stromal cells, and fibroblasts comprise most of the nonimmune microenvironment in lymphoma, contributing to tumorigenicity. Research on nonimmune microenvironmental components in AIDS-related NHL is mostly focused on neovascularization, although whether HIV1 can alter this process by productive infection of endothelial cells is still debated.^227^ Although aberrant neovascularization is a typical occurrence in various viral diseases, the precise mechanism of viral carcinogenesis in these environments may be crucial for the treatment of HIV-associated lymphoma. Therefore, diagnosis and treatment are very important.^228^

## 3 Antiviral Strategies

**Figure 4:**
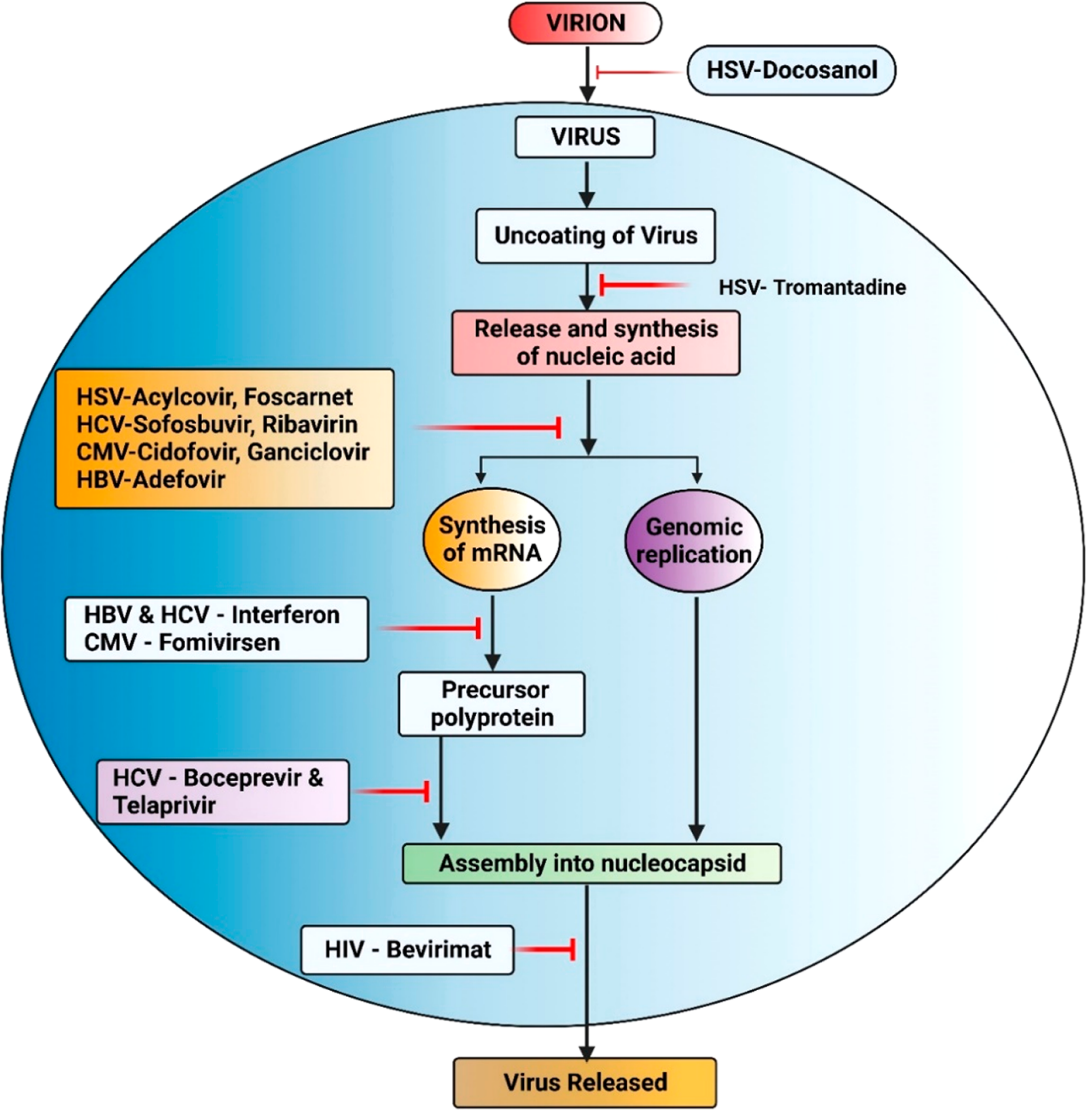
Different antiviral agents to curb the action of oncogenic viruses on the molecular front (Created by Biorender).

Viral propagation involves exploiting and subverting the cellular machinery of the host through various tactics. The administration of antivirals is vital for reducing the viral load and, thus, propagating the host toward recovery. Antivirals can be derived from natural or chemical sources with varying specificity toward viral strains. In patients with oncological malignancies with prominent or latent hepatitis B or C infection, a nucleoside analogue antiviral therapy successfully prevents virus reactivation. Hepatitis B Ag-positive patients have also reported regression of lymphoma following successful anti-HBV treatment with lamivudine and entecavir.^239^ Next, the potential of human papillomavirus to interfere with the proper functioning of the interferon response based on several molecular pathways coordinated by human papillomavirus proteins aimed to prevent infection clearance results in the creation of an immunotolerant environment that promotes the establishment of persistence and cancer. For instance, chemically derived Imiquimod is an immunomodulatory dosage that affects both the innate and adaptive immune responses and the activation of natural killer cells, the production and release of interferon (IFN)-alpha, tumor necrosis factor-alpha, and interleukin-12, in addition to the suppression of viral replication.^240^ Cidofovir is another nucleotide analogue that works by inhibiting viral DNA polymerase, and the medication cidofovir also helps to reduce the HPV-positive tumor cells’ propensity for metastasis.^241^ Other chemically derived antivirals, such as Ganciclovir, Acyclovir, and Famciclovir, target the viral DNA of the Epstein–Bar virus during the infection stage, thus preventing its progression along with the inhibition of host cell DNA polymerase (Figure 4). In the case of Merkel Cell Polyomavirus infection, a monoclonal antiviral, namely Nivolumab, targets Programmed Cell Death Protein 1 with an eventual inhibitory effect on the hyperactive T cells. Likewise, Ipilimumab targets the CTLA-4 protein causing detrimental defects in the cell repair machinery.

Similarly, natural compounds, such as those found in herbal pharmaceuticals, are also used to search for new antiviral agents. The expression of E6 and E7 viral oncoproteins of human papillomaviruses has the primary role in cervical carcinoma.^242^ The primary component of C. longa, curcumin, has an inhibitory effect on the expression of these E6 and E7 genes of two different kinds of HPVs, which are highly oncogenic. Andrographolide, neoandrographolide, dehydro-andrographolide, and several natural and synthetic derivatives possess notable antiviral activity against HIV, influenza A, HBV and HCV without any significant cytotoxic effect at virus-inhibiting concentrations. In addition, naturally derived antivirals from *Detarium microcarpum*, *Bupleurum kaoi*, and others help to inhibit viral entry of HCV. Similarly, an antiviral obtained from *Eclipta alba*, *Taraxacum officinale*, is known to inhibit HCV’s NS5B replicate activity. Table 2 provides a comprehensive list of antivirals from both chemical and natural sources.

**Table 2:** A List of Antivirals Obtained Either Chemically or Naturally That Have Varied Activity against Several Oncogenic Viruses

A. Chemical Antivirals				
Viruses	Antivirals	Functions	Target	References
Epstein–Barr virus	Ganciclovir	Prevent the progression of viral DNA, inhibits host cell DNA polymerase	Viral DNA	(3)
	Acyclovir			
	Famciclovir			
	Rituximab (Monoclonal antibody)	Targets all B cells expressing CD20 (not specifically target cells containing EBV)	B cells	
	Rapamycin (Immunosuppressive agent)	Decrease tumor growth and metastasis in a mouse model of EBV-associated oncogenesis	P13K Pathway	
	Valganciclovir + Alemtuzumab	Suppresses replication of EBV	Reactivation of EBV by targeting CD52 on B- and T-lymphocyte)	(248−250)
	Maribavir	Inhibits the EBV protein kinases	Viral DNA	(251)
	Cidofovir	Inhibitors of murine herpesvirus replication and inhibits ribonucleotide reductase (RR)	Phosphodiesterase and EBV-transformed epithelial cells/Xenografts	(249,251)
	Rutamarin	Inhibiting replication of viral DNA	Topoisomerase II	(251)
Hepatitis B and C virus	Lamivudine	HBV DNA chain termination	Reverse transcriptase	(252)
	Adefovir	Inhibits HBV DNA synthesis	Reverse transcriptase	(252,253)
	Entecavir	Inhibits all steps in the HBV viral replication	HBV polymerase	(254,255)
	Telbivudine	Inhibits HBV DNA chain termination and viral replication	Second strand of DNA and HBV DNA polymerase (reverse transcriptase)	(256−258)
	Tenofovir		Reverse transcriptase	(259)
	Boceprevir	Inhibits viral HCV replication as well as disrupts the processing of viral proteins	HCV nonstructural 3/4A protease inhibitors	(252)
	Telaprevir			
	Sofosbuvir	Executes chain termination and prevents viral replication of HCV	Inhibits HCV NS5B (nonstructural protein 5B) RNA-dependent RNA polymerase	(260)
	Simeprevir	Blocks the function of adapters protein	Polyprotein of HCV	(261)
	Faldaprevir	Cleaves the HCV-encoded polyprotein	NS3/4A protease	(262)
	Ribavirin	Depletion of intracellular GTP and increased hepatitis C virus mutagenesis	ITP pyrophosphatase (ITPase)	(263)
	Asunaprevir	Inhibits viral replication	HCV NS3 protease	(264)
	Danoprevir	Prevents the cleavage and processing of HCV viral proteins	HCV NS3/4A protease	(265)
	Vaniprevir	Inhibits the enzymatic activity	HCV NS3/4A protease	(266)
	Daclatasvir	Blocks both virion assembly/secretion in vivo and intracellular viral RNA synthesis	HCV NS5A proteins	(267)
	Ledipasvir	Prevents hyperphosphorylation of proteins for viral production		(268)
	Ombitasvir	Blocking signaling interactions and modification of the HCV replication complex		(269,270)
Human Papillomavirus	Cidofovir	Converted to its active form as triphosphorylated cidofovir and is likely to induce chain termination	HPV DNA	(271,272)
	GS-9191	Inhibits DNA synthesis	DNA polymerase α and ß	(273)
	ODE-Bn-PMEG/ABI-1968	Inhibits HPV origin-dependent plasmid amplification	HPV DNA	(274)
	Acyclovir	Inhibits the integration of viral DNA and the replication of cellular DNA	HPV DNA	(275)
	Imiquimod	Stimulates the innate and acquired immune responses followed by apoptosis of infected tissues	Toll-like receptors	(276)
	Cimetidine	Improves cellular immunity and wart remission	Stimulates Th1 cells to catalyze interleukins (IL)-2, IL-12, tumor necrosis factor (TNF)-α, and interferon (IFN)-γ	(276)
	Bleomycin	Eliminates pyrimidine and purine bases and affects cellular DNA synthesis	Cellular DNA	(277)
Merkel Cell Polyomavirus	Pembrolizumab	Counteracts its interaction with its known ligands	Programmed cell death protein 1	
	Avelumab	1. Blocks the interaction between PD-L1 and its receptors PD-1 and B7.1	Programmed cell death 1, Ligand 1	(278,279)
		2. Stimulates ADCC in addition to immune checkpoint inhibition		
	Nivolumab	Prevents PD-L1 and PD-L2 from inhibiting the action of T cells	Programmed cell death protein 1	(280)
	Ipilimumab	Defects in the DNA repair machinery	CTLA-4 protein	(281)
Kaposi Sarcoma Herpesvirus	Cidofovir	Inhibitors of murine herpesvirus replication and inhibits ribonucleotide reductase (RR)	Phosphodiesterase and EBV-transformed epithelial cells/Xenografts	(249)
	Ganciclovir	1. Prevents the progression of viral DNA, inhibits host cell DNA polymerase	Viral DNA	(3,282)
		2. Reduces plasma viral load of KSHV and can prevent KS in KSHV-seropositive transplant recipients		
	Bortezomib	Stabilizes the cellular proteins involved in suppressing cellular proliferation and promotes apoptosis, including p21, p27, p53, and I kappa-B	Human 26 S proteasome	(283)
	Tocilizumab (Antibody)	Blocks membrane-bound and soluble gp80 signal transduction	gp80	(284)
	Siltuximab (Monoclonal antibody)	Inhibits binding to soluble and membrane-bound IL-6 receptors	Interleukin 6	(285)
	Pomalidomide	Induces and elevates the B7–2 and ICAM-1 expression in PEL cells	Activation of T cells and NK cell-mediated killing of PEL cells	(286)
	Bevacizumab (Monoclonal antibody)	Exerts its effects by binding and inactivating serum VEGF and is unable to interact with its cell surface receptors, thereby proangiogenic signaling is terminated	VEGF-A, subcomponents subunits (A, B, C), and Affinity Immunoglobulin Fc gamma receptors	(286,287)
	Doxorubicin	Complexes are formed with DNA by intercalation between base pairs, and it inhibits topoisomerase II action by stabilizing the complex of DNA–topoisomerase II	DNA topoisomerase 2-alpha and DNA	(288)
Human Immunodeficiency Virus (HIV)	Saquinavir + Ritonavir	Hydroxyethylene scaffold mimics the HIV protease for cleaving, thereby carrying out the proteolysis of Gag polyprotein and producing immature and noninfectious particles	HIV protease	(289−292)
	Lopinavir			
	Indinavir			
	Nelfinavir			
	Amprenavir			
	Atazanavir			
	Tipranavir			
	Darunavir + Ritonavir	Prevents viral replication	HIV protease	(293,294)
	Lamivudine	Prevents the formation of phosphodiester bonds between the Nucleotide Reverse Transcriptase Inhibitors and nucleoside triphosphate	Reverse transcriptase	(295−297)
	Zidovudine			
	Abacavir			
	Didanosine			
	Emtricitabine			
	Stavudine			
	Zalcitabine			
	Tenofovir disoprovil fumarate			
	Entravirine	Non-Nucleoside Reverse Transcriptase Inhibitor changes the spatial conformation of the substrate binding site and reduces the polymerase activity	Reverse transcriptase	(298)
	Delavirdine			
	Efavirenz			
	Nevirapine			
	Rilpivirine (Phase 3)			
	Raltegravir	1. Binds between the integrase and viral DNA to a specific complex	Integrase (Strand transfer reaction)	(299−301)
	MK-0518	2. Interaction takes place between the inhibitor and Mg metal ion in the active site of integrase as well as the DNA		
	Elvitegravir			
Human T-lymphotropic Virus (HTLV)	Alemtuzumab	Blocks the TAC antigen	ATLL cells and HTLV1-infected cells	(302)
	Arsenic trioxide + Interferon-α	Induces cell cycle inhibition and apoptosis	HTLV1-infected cells and malignant ATLL cells	(303,304)
	Lamivudine	Can inhibit viral replication in HTLV1-infected cells in single-use or combination therapy	Nucleoside analogs	(305,306)
	Abacavir			
	Zidovudine			
	Didanosine			
	Emtricitabine			
	Stavudine			
	Zalcitabine			
	Tenofovir disoprovil fumarate			
	Raltegravir	Potent to inhibit strand transfer reaction and number of integration of events directly	HTLV1 Integrase	(307)
	Romidepsin	1. Can suppress the expression of NF-κB and AP-1	ATLL cells and HTLV1-infected cells	(308,309)
		2. Induces apoptosis of HTLV-1 infected cells and ATLL cells		
	Niclosamide	Induces degradation in protein and inhibits viral gene transcription of HTLV1	Tax protein	(310)
	Chondroitin Sulfate Type E	Interacts with the recombinant envelope protein of the virus at the C-terminus and blocks the binding of the virus to the human T cell	Human T cell	(311)
	Darunavir	Prevents viral replication	HTLV1 Protease	(312)
	Arsenic trioxide + Interferon-α	Induces cell cycle inhibition and apoptosis	HTLV1-infected cells and malignant ATLL cells	(303,304)

B. Natural Antivirals			
Antivirals	Function	Virus/Target	References
*Terminalia bellerica*	Inhibits viral entry, replication, and maturation of HBV particles	Hepatitis B virus	(313−315)
*Phyllanthus Amarus*			
*Hybanthusenneaspermus*			
*Enicostemmaaxillare*			
*Bombyx mori L*			
*Boehmeria nivea*			
*Trichiliadregeana*	Inhibits viral entry	Hepatitis C virus	
*Detarium microcarpum*			
*Phragmanthera capitata*			
*Bupleurum kaoi*			
Anthocyanidin			
*Alloeocomatellapolycladia*	Suppression of the helicase activity of HCV NS3		
*Fusarium equiseti*	Inhibition of HCV NS3/4A protease		
*Eclipta alba*	Inhibition of HCV NS5B replicase activity		
*Taraxacum officinale*			
*Swietenia macrophylla*	Reduction of HCV protein and HCV–RNA levels		
*Entada africana*	Broad antiviral activity		
Grape seed	Suppression of HCV-induced Cox-2		
Flavanone	Release/Assembly		
*Curcuma longa*L	Inhibits immediate–early gene expression	Herpes simplex virus 1	
*Houttuynia cordata*	Blocks viral binding and suppresses NF-κB activation	Herpes simplex virus 1	
		Herpes simplex virus 2	
*Pistacia vera L*	Inhibits expression of HSV-1 viral proteins and viral DNA synthesis	Herpes simplex virus 1	
*Prunus dulcis*	Blocks viral binding		
*Aloe Vera*	Reduction of cytopathic effect (CPE)		
*Cephalotaxaceae*	Targets the cellular factor eIF4E		
*Curcuma longa L*	Downregulating expression of oncogenes E6 and E7	HPV-16	
		HPV-18	
*Ficus carica*		HPV-16	
*C. longa, A. indica*, *E. officinalis*, *A. vera*	Prevents the entry of HPV 16 in Hela cells		
*Green tea*	Promoting apoptosis and inhibiting cellular transcriptional factors		
*Pinelliapedatisecta*		HPV-16	
		HPV-18	
*Cudraniatricuspidata*		HPV-16	
*Curcuma longa L*		HPV-18	
		HPV-16	
*Bryophyllum pinnata*		HPV-18	
*Phyllanthus emblica*		HPV-16	
		HPV-18	
*Kaempferia parviflora*		HPV-16	
*Podophyllum*	Mitotic arrest in cell cycle	Genital warts	(316)
*Nostoc ellipsosporum*	Breaching inhibitors	HIV	(313−315)
*Griffithsia* sp.			
*Stellettaclavosa*			
*Siliquariaspongia mirabilis*			
*Syzygiumclaviflorum*	Maturation inhibitors		
*Synthetic derivative of betulinic acid*			
*Rheum palmatum*	Integrase inhibitors and reverse transcriptase		
*Morus nigra*			
*Justica gendarussa*			
*Calophyllumlanigerum*			
*Euphorbia kansui*	Latency-reversing agents		
*Theobroma cacao*			
*Andrographis paniculata*	Inhibits transcription of IE genes in EVB and the production of virions	Epstein–Barr Virus	(249)
*Polygonum cuspidatum*	Inhibits the lytic cycle of EBV		
*Saururus chinensis*	Inhibits replication of EBV lytic cycle		
*Rhus chinensis*	Reduces the number of EBV particles		
*Thelypteris torresiana*	Inhibiting the EBV virus lytic cycle		
*Psoralea corylifolia*	Inhibits early steps of the replicative lytic cycle in EBV		
*Angelica archangelica*			
Borrelidin	Inhibits tRNA synthesis	Merkel Cell Carcinoma	(317)
Mechlorethamine	Causes DNA damage		
Plumbagin	ROS/redox-active, proteasome		
Mitomycin C	Causes DNA damage		
Cladribine	Inhibits DNA synthesis		
Clofarabine			
Pyrrolidine dithiocarbamate	ROS, proteasome, NFκB		
Etoposide	Topoisomerase II/causes DNA damage		
Gloxazone	Inhibits DNA synthesis		
Panobinostat	Histone deacetylase inhibitors/ROS active		
Disulfiram	ROS, proteasome, NF-κB		
Thapsigargin	Stress on the endoplasmic reticulum		
Englerin A	Protein Kinase C		
Furoxanobenzofuroxan	Inhibits monoamine oxidase		
5-Nitroso-8-quinolinol	Histone deacetylase inhibitors/ROS active		
*Phytolacca americana*	Depurinates nucleotides/feedback inhibition of HTLV-I gene expression	Human T-lymphotropic virus 1	(318)
*Scutellaria baicalensis georgi*	Inhibits reverse transcriptase activity in HTLV-I-infected cells		(319)
Curcumin metabolites (Curcumin-sulfate, curcumin-glucuronide, and tetrahydro-curcumin)	Potent HTLV1 protease inhibitors		(320)
Oleandrin	Inhibits virological synapse formation		(321)

These chemicals and naturally derived antiviral drugs have been reported and shown their potential for viral oncogenesis therapies (Figure 4). Several studies have reported on the effectiveness of antiviral drugs in preventing oncogenesis. In 2008, a study by Yoshizaki et al. reported the antitumor potential of cidofovir against nasopharyngeal carcinoma (NPC). Two patients whose earlier multi-round therapy was treated with cidofovir once every three weeks (75 mg/mL solution diluted to 15 mg/mL just before injection) showed a reduction in the tumor cell population.^243^ In 2017, Shaimerdenova et al. studied the effect of the antiviral agent acyclovir on the MCF7 breast cancer cell line. The study reported that acyclovir treatment decreases cancer cell proliferation, colony formation ability, and cell invasion capacity by up-regulating Caspase-3 and down-regulating aldehyde dehydrogenase (ALDH) activity.^244^ Mattson et al. reported in 2009 that zidovudine treatment combined with the chemotherapeutic agent cisplatin had increased the apoptosis level of head and neck cancer cells. The combination synergistically triggers abnormal regulation of the mitochondria by increased oxidative stress response and significant cytotoxic effect on the cancer cell through inhibiting thiol metabolism.^245^ Recently, Huang et al. in 2021 reported the differential effect of oseltamivir in inducing liver cancer cell death both in vitro and in vivo. Treatment with oseltamivir decreased migration and invasion of liver cancer cells and increased autophagy in Huh-7 cells.^246^ Urtishak et al. also reported that the antiviral drug ribavirin decreased the elevated level of EIF4E protein in most cases of infant acute lymphoblastic leukemia (ALL).^247^ In this study it has been reported that treating ribavirin by actively dividing infant ALL cells into bone marrow stromal cells (BMSCs) at clinically achievable concentrations decreases oncogenic EIF4E-regulated cell growth and survival proteins.

## 4 Recent Progress on Drug Developments for Oncogenic Virus

The existing challenges toward developing new drugs to prevent virus-associated cancer have heated up in recent years. Much more research is being conducted to have some positive outcomes that could be translated. For instance, the NCT03783078 trial primarily investigated pembrolizumab as a first-line therapy for advanced Merkel cell carcinoma. Pembrolizumab falls under the category of a monoclonal antibody that obstructed the interaction of programmed death receptor-1 (PD-1) with programmed death ligand-1(PD-L1) and PD-L2, thereby inhibiting antitumor immune response.^322^ Another trial NCT01472263 has primarily focused on the safety interventional studies of the drug Pentoxifylline in HTLV1 patients. Pentoxifylline falls under the category of methylxanthine molecules that inhibit the action of phosphodiesterase type IV, which in turn delays the degradation process of cAMP and prostanoids. The activity of NF-κB and NF-AT transcription factors gets disrupted with the high influx of intracellular cAMP, eventually inhibiting TNF-α production, thus deregulating the action of HTLV1.^323^

The NCT02346227 randomized placebo-controlled trial essentially puts weight on the safety studies of AV2 for HPV-associated lesions of the uterine cervix. A complex combination of naturally available essential oils, namely, carvone, eugenol, geraniol, and nerolidol, constitutes an antiviral formulation named Antiviral 2 developed by Cesa Alliance, Luxembourg. The essential oils have already received FDA approval, and their antiviral effect is well documented. As per reports, antiviral 2 formulations have been shown to inhibit viral replication by degenerating HPV-induced cervical lesions.^324,325^ This NCT00002445 trial primarily focuses on the safety and effectiveness of intranasal-based IM862 drug formulation in treating Kaposi’s Sarcoma/HHV-8 in AIDS Patients. Chemically, IM862 is derived as a dipeptide from L-glutamyl-L-tryptophan. IM862 helps to inhibit tumor angiogenesis, which gives rise to new blood vessels that provide a redundant nutrition supply to the tumor cells. The action of IM862 is executed through the upregulation of natural killer cell activity and the inhibition of vascular endothelial growth factor.^326^ The NCT02640482 primarily investigates ABT-493 efficacy and safety of ABT-493/ABT-530 in adults with chronic HCV Type 2 infection. ABT-493 is an antagonist of the HCV nonstructural (NS) protein 3/4A protease, while ABT-530 is an antagonist of the HCV NS5A protease. These substances exhibit strong in vitro-based antiviral efficacy against all key HCV genotypes.^327^ One more instance of progress has been reported where NCT00805675 primarily focuses on the effects of telbivudine and tenofovir disoproxil fumarate treatment for HBV DNA. As it is a transparent, passively regulated viral kinetics study, the participant and the physician would know the investigative medicine administered to each patient. Telbivudine is classified as an L-nucleoside that exhibits structural similarity with lamivudine. The action of telbivudine is highly specific in nature as it targets the HBV DNA synthesis process without disrupting human DNA or other viral strains.^328,329^ Some other drug clinical trials for targeting the oncogenic viruses are listed in Table 3.

**Table 3:** Completed Clinical Trials Conducted for Drugs Relevant to the Following Oncogenic Viruses^a^

S. No.	Title	Intervention	Outcome measures	Sponsors/ Collaborators	Phase	Enrollment	NCT Number
**(i) MCPyV**							
1.	Pembrolizumab (MK-3475) as First-line Therapy for Advanced Merkel Cell Carcinoma (MK-3475-913)	Drug: Pembrolizumab (MK-3475)	Objective Response Rate (ORR), Duration of Response (DOR), Progression-free Survival (PFS), Overall Survival (OS), Number of Participants with One or More Adverse Events (AEs), Number of Participants Who Discontinued from Study Treatment Due to an AE	Merck Sharp & Dohme LLC	Phase 3	55	NCT03783078 (https://ClinicalTrials.gov/show/NCT03783078, Accessed on 22 March 2023)
2.	Testing Pembrolizumab Versus Observation in Patients with Merkel Cell Carcinoma After Surgery, STAMP Study	Other: Best Practice, Procedure: Biospecimen Collection, Procedure: Computed Tomography, Biological: Pembrolizumab, Procedure: Positron Emission Tomography, Radiation: Radiation Therapy	Recurrence-free survival (RFS), OS, Impact of radiation therapy on RFS, Impact of radiation therapy on OS, Impact of radiation therapy on distant metastasis-free survival (DMFS), Incidence of adverse events	National Cancer Institute (NCI)	Phase 3	280	NCT03712605 (https://ClinicalTrials.gov/show/NCT03712605, Accessed on 22 March 2023)
3.	Adjuvant Avelumab in Merkel Cell Cancer	Drug: Avelumab, Other: Peripheral Blood Collection, Other: Placebo	Relapse-free survival, Disease-specific survival, Distant-metastases free survival, Incidence of adverse events, Overall survival	University of Washington, EMD Serono	Phase 3	100	NCT03271372 (https://ClinicalTrials.gov/show/NCT03271372, Accessed on 22 March 2023)
4.	Evaluating Length of Treatment With PD-1/PD-L1 Inhibitor in Advanced Solid Tumors	Drug: Continue PD-1/PD-L1 Inhibitors treatment; other: Discontinue PD-1/PD-L1–1 inhibitor	Time to next treatment, Progression-free Survival (PFS) (at between 2 and 3.9 months), Progression-free Survival (PFS) (at between 4 and 7.9 months), Progression-free Survival (PFS), Incidence of irAEs (Immune-Related Adverse Events), Overall Survival (OS), Best Objective Response (BOR)	Jason J. Luke, MD, University of Pittsburgh	Phase 3	578	NCT04157985 (https://ClinicalTrials.gov/show/NCT04157985, Accessed on 22 March 2023)
5.	Study Comparing the Standard Administration of IO Versus the Same IO Administered Each 3 Months in Patients with Metastatic Cancer in Response After 6 Months of Standard IO	Drug: Reduced dose intensity of IO	Progression-free survival (PFS), Cost-effectiveness analysis of the proposed therapeutic strategy, Immune progression-free survival (iPFS), Objective response rate (ORR), Overall survival (OS), Duration of response (DoR), Quality of life questionnaire - Core 30 (QLQ-C30), The Developed 5-level version of EQ-5D (EQ-5D-5L) questionnaire, Hospital anxiety and depression scale (HADS), Fear of relapse questionnaire, Safety profile	UNICANCER	Phase 3	646	NCT05078047 (https://ClinicalTrials.gov/show/NCT05078047, Accessed on 22 March 2023)
**(ii) HTLV1**							
1.	Use of Pentoxifylline in Human T-lymphotropic Virus Type-1 (HTLV-1) Diseases	Drug: Pentoxifylline, Drug: Placebo	Functional neurological capacity, Reduced in cytokines and chemokines	Hospital Universitario Professor Edgard Santos	Phase 3	48	NCT01472263 (https://ClinicalTrials.gov/show/NCT01472263, Accessed on 22 March 2023)
2.	Zidovudine Plus Lamivudine in HTLV-I-associated Myelopathy: A Randomized Trial	Drug: Zidovudine/lamivudine, Drug: Placebos	Timed walk, Osame’s Motor Disability Score, Pain score, Urinary frequency, HTLV-1 proviral load, CD25%, HLA-DR%	Imperial College London	Phases 2 and 3	16	NCT00272480 (https://ClinicalTrials.gov/show/NCT00272480, Accessed on 22 March 2023)
3.	Ciclosporin in HTLV-1 Associated Myelopathy/Tropical Spastic Paraparesis (HAM/TSP)	Drug: Ciclosporin	Number of Patients with Lack of Objective Clinical Improvement, Change in Timed Walk Sl. No. Between Baseline and 12 Weeks	Imperial College London, Medical Research Council, University Hospital Birmingham, Imperial College Healthcare NHS Trust	Phases 2 and 3	7	NCT00773292 (https://ClinicalTrials.gov/show/NCT00773292, Accessed on 22 March 2023)
4.	Use of Valproic Acid to Treat Tropical Spastic Paraparesis/HTLV-1-Associated Myelopathy (TSP/HAM)	Drug: Valproic acid, Drug: Corticosteroids, Drug: valproic acid plus corticosteroids	Neurological scales, Quality of life	University of Sao Paulo	Phase 3	60	NCT00681980 (https://ClinicalTrials.gov/show/NCT00681980, Accessed on 22 March 2023)
5.	Pilot Study of Combination Therapy With CHOP-Zenapax (CHOP-daclizumab)	Drug: CHOP-daclizumab		King’s College Hospital NHS Trust	Phase 4		NCT01418430 (https://ClinicalTrials.gov/show/NCT01418430, Accessed on 22 March 2023)
**(iii) HPV**							
1.	Impact of AV2 Antiviral Drug on the Treatment of HPV-associated Lesions of the Uterine Cervix	Drug: AV2, Drug: Placebo	Change of lesions, absence of HPV DNA, the correlation between change of lesions and change in HPV DNA, Change in HPV viral particle load	Jean-Pierre Van Geertruyden, University of Kinshasa, University Hospital, Antwerp, Universiteit Antwerpen	Phase 3	327	NCT02346227 (https://ClinicalTrials.gov/show/NCT02346227, Accessed on 22 March 2023)
2.	Recombinant Human Interferon a-2b Gel for HPV Gynaecological Infections	Drug: Yallaferon	The difference in hr-HPV DNA negative conversion rate, Secondary efficacy end points were the differences of single-type HPV infection, dual infection, and multiple infections in the sixth month between the two groups	Lee’s Pharmaceutical Limited	Phases 2 and 3	325	NCT01824992 (https://ClinicalTrials.gov/show/NCT01824992, Accessed on 22 March 2023)
3.	Study of the Efficacy and Safety of the Drug Ingaron (Interferon-gamma) in the Treatment of Anogenital Warts	Drug: Interferon-gamma human recombinant (IFN-G)	Recurrence of anogenital warts	SPP Pharmaclon Ltd.	Phase 3	30	NCT05156541 (https://ClinicalTrials.gov/show/NCT05156541, Accessed on 22 March 2023)
4.	Delivery, Uptake and Acceptability of HPV Vaccination in Tanzanian Girls	Biological: Gardasil HPV vaccine	Vaccine coverage by delivery strategy, Vaccine coverage (dose 2) by delivery strategy, Vaccine coverage (dose 1) by delivery strategy, Factors associated with refusal to vaccinate or to complete vaccination course, Identification of barriers to HPV vaccination, Estimation of the costs of introducing and scaling up HPV vaccines in schools	London School of Hygiene and Tropical Medicine, National Institute for Medical Research, Tanzania, Ocean Road Cancer Institute, Tanzania, Institut Catalan d’ Oncologia, Spain, Medical Research Council Social & Public Health Sciences Unit, UK, International Union Against Cancer, Switzerland	Phase 4	5532	NCT01173900 (https://ClinicalTrials.gov/show/NCT01173900, Accessed on 22 March 2023)
5.	Immunogenicity and Safety of Dengue Tetravalent Vaccine (TDV) and Recombinant 9-valent Human Papillomavirus Vaccine (9vHPV) in Participants Aged 9 to < 15 Years	Biological: 9vHPV vaccine, Biological: Dengue Tetravalent Vaccine (TDV)	Geometric Mean Titers (GMTs) for Human Papillomavirus (HPV) Types 6, 11, 16, 18, 31, 33, 45, 52, and 58, Percentage of Participants with Seropositivity for HPV Types 6, 11, 16, 18, 31, 33, 45, 52, and 58 as Measured by Immunoglobulin G Binding Assay (IgGBA) or Equivalent Assay, GMTs of Neutralizing Antibodies for Each of the 4 Dengue Serotypes, Percentage of Participants with Seropositivity for Each of the 4 Dengue Serotypes, Percentage of Participants with Seropositivity for Multiple (2, 3, or 4) Dengue Serotypes, Percentage of Participants with Solicited Local Reactions for 7 Days Following Vaccination by Severity, Percentage of Participants with Solicited Systemic Adverse Events (AEs) for 14 days Following Vaccination by Severity, Percentage of Participants with any Unsolicited AEs for 28 days Following Vaccination, Percentage of Participants with Serious Adverse Events (SAEs)	Takeda	Phase 3	618	NCT04313244 (https://ClinicalTrials.gov/show/NCT04313244, Accessed on 22 March 2023)
6.	Safety and Immunogenicity of Human Papillomavirus (HPV) Vaccine in Solid Organ Transplant Recipients	Biological: Human papillomavirus quadrivalent vaccine	The primary outcome will be a 2-fold rise in the type-specific HPV titer for at least one of the four serotypes contained in the vaccine at month 7, Vaccine adverse events, including episodes of rejection up to 1 year after study enrolment, Immunogenicity at 36 months postvaccination.	University of Alberta	Phase 3	50	NCT00677677 (https://ClinicalTrials.gov/show/NCT00677677, Accessed on 22 March 2023)
7.	Immunogenicity and Safety of a Quadrivalent Human Papillomavirus (HPV) Vaccine in Patients With SLE: a Controlled Study	Drug: human papillomavirus vaccination (Gardasil)	Antibody titers against 4 strains of human papillomavirus	Tuen Mun Hospital	Phase 4	100	NCT00911521 (https://ClinicalTrials.gov/show/NCT00911521, Accessed on 22 March 2023)
**(iv) KSHV**							
1.	Safety and Effectiveness of an Experimental Drug, IM862, in Treating Kaposi’s Sarcoma in AIDS Patients	Drug: IM862		Cytran, NIH AIDS Clinical Trials Information Service	Phase 3	200	NCT00002445 (https://ClinicalTrials.gov/show/NCT00002445, Accessed on 22 March 2023)
2.	Antiretroviral Therapy (ART) Alone or With Delayed Chemo Versus ART With Immediate Chemo for Limited AIDS-related Kaposi’s Sarcoma	Drug: efavirenz/emtricitabine/tenofovir disoproxil fumarate, Drug: etoposide	Kaposi Sarcoma (KS) Status at Week 48 Compared to Study Entry, KS Progressive Disease at Week 48 Compared to Study Entry	AIDS Clinical Trials Group, National Institute of Allergy, and Infectious Diseases (NIAID)	Phase 3	192	NCT01352117 (https://ClinicalTrials.gov/show/NCT01352117, Accessed on 22 March 2023)
3.	HIV/AIDS Kaposis Sarcoma: Comparison of Response to HAART vs HAART Plus CXT	Drug: Generic HAART Triomune: d4T, 3TC, NVP, Drug: Generic HAART Triomune: d4T, 3TC, NVP, and chemotherapy ABV	Clinical response of KS, Skin: tumor measurements of 5 indicator skin lesions. Assessment of KS as per AMC RKS 02 (www.amc.uab.edu), photography of indicator lesions with metric tape in the frame, Visceral: chest radiograph and endoscopy, where necessary, bronchoscopy, Safety and toxicity by DAIDS Toxicity criteria, Immunological and virological response to HAART as measured by CD4 and HIV-viral load, QOL by EORTC QLQ C30, Adherence	University of KwaZulu, AIDS Care Research in Africa, National Research Foundation, Singapore, AIDS Malignancy Consortium, Cipla Medpro, Dermatological Society of South Africa	Phase 4	112	NCT00380770 (https://ClinicalTrials.gov/show/NCT00380770, Accessed on 22 March 2023)
4.	Use of Stealth Liposomal Doxorubicin HCl (DOX-SL) in the Treatment of Moderate to Severe AIDS-Related Kaposi’s Sarcoma.	Drug: Doxorubicin hydrochloride (liposomal)		Sequus Pharmaceuticals, NIH AIDS Clinical Trials Information Service	Phase 3		NCT00002147 (https://ClinicalTrials.gov/show/NCT00002147, Accessed on 22 March 2023)
5.	A Pilot Study of the Effects of Highly Active Antiretroviral Therapy on Kaposi’s Sarcoma in Zimbabwe	Drug: abacavir/3TC/zidovudine, Drug: abacavir /3TC plus ritonavir boosted lopinavir	Compare effects of twice-daily all-(NRTI) antiretroviral regimen to a once-daily regimen of 2 NRTIs plus a protease inhibitor AIDS-KS subjects with good virologic suppression on all-NRTI regimen.	Parirenyatwa Hospital, University of Colorado, Denver, GlaxoSmithKline, Abbott	Phases 2 and 3	49	NCT00834457 (https://ClinicalTrials.gov/show/NCT00834457, Accessed on 22 March 2023)
6.	A Study of DOX-SL in the Treatment of AIDS-Related Kaposi’s Sarcoma	Drug: Doxorubicin hydrochloride (liposomal)		Sequus Pharmaceuticals, NIH AIDS Clinical Trials Information Service	Phase 3		NCT00002319 (https://ClinicalTrials.gov/show/NCT00002319, Accessed on 22 March 2023)
7.	Paclitaxel Compared with Doxorubicin in Treating Patients with Advanced AIDS-Related Kaposi’s Sarcoma	Drug: paclitaxel, Drug: pegylated liposomal doxorubicin hydrochloride, Other: laboratory biomarker analysis, Procedure: quality-of-life assessment	Progression-free survival, Patients’ health-related quality of life (QOL) in terms of change in pain score, e Antibody titers edema-related mobility, gastrointestinal (GI) symptoms and respiratory symptoms based on the total score from the Functional Assessment of HIV Infection (FAHI) v3	National Cancer Institute (NCI)	Phase 3	240	NCT00003350 (https://ClinicalTrials.gov/show/NCT00003350, Accessed on 22 March 2023)
8.	Randomized, Comparative Trial of DOX-SL (Stealth Liposomal Doxorubicin Hydrochloride) Versus Bleomycin and Vincristine in the Treatment of AIDS-Related Kaposi’s Sarcoma	Drug: Doxorubicin hydrochloride (liposomal), Drug: Bleomycin sulfate, Drug: Vincristine sulfate		Sequus Pharmaceuticals, NIH AIDS Clinical Trials Information Service	Phase 3	220	NCT00002105 (https://ClinicalTrials.gov/show/NCT00002105, Accessed on 22 March 2023)
9.	Doxorubicin in Treating Patients With AIDS-Related Kaposi’s Sarcoma	Drug: daunorubicin hydrochloride, Drug: pegylated liposomal doxorubicin hydrochloride		Roswell Park Cancer Institute	Phase 3		NCT00002985 (https://ClinicalTrials.gov/show/NCT00000994, Accessed on 22 March 2023)
10.	A Randomized Phase III Clinical Trial of Daunoxome Versus Combination Chemotherapy with Adriamycin/Bleomycin/Vincristine (ABV) in the Treatment of HIV-Associated Kaposi’s Sarcoma.	Drug: Daunorubicin (liposomal), Drug: Bleomycin sulfate, Drug: Vincristine sulfate, Drug: Doxorubicin hydrochloride		Nexstar Pharmaceuticals, NIH AIDS Clinical Trials Information Service	Phase 3		NCT00002093 (https://ClinicalTrials.gov/show/NCT00000994, Accessed on 22 March 2023)
11.	A Study of AZT in HIV-Infected Patients With AIDS-Related Kaposi’s Sarcoma	Drug: Zidovudine		National Institute of Allergy and Infectious Diseases (NIAID)	Phase 3	240	NCT00000994 (https://ClinicalTrials.gov/show/NCT00000994, Accessed on 22 March 2023)
12.	Anti-Retrovirals for Kaposi’s Sarcoma	Drug: Lopinavir/ritonavir plus Emtricitabine/Tenofovir versus Efavirenz plus Emtricitabine/Tenofovir	Blinded assessment of the change in the burden of KS lesions, CD4+ T cell count and HIV plasma HIV RNA levels, KSHV DNA levels in saliva and blood, Humoral and cellular KSHV immune response markers, Quality-of-life assessment, Incidence of Kaposi’s sarcoma-associated Immune Reconstitution Inflammatory Syndrome (KS-IRIS)	University of California, San Francisco, National Institutes of Health (NIH), Gilead Sciences, Abbott, Merck Sharp & Dohme LLC	Phase 4	224	NCT00444379 (https://ClinicalTrials.gov/show/NCT00444379, Accessed on 22 March 2023)
**(v) HCV**							
1.	A Study to Evaluate the Efficacy and Safety of ABT-493/ABT-530 in Adults with Chronic Hepatitis C Virus (HCV) Genotype 2 Infection	Drug: ABT-493/ABT-530, Drug: Placebo for ABT-493/ABT-530	Percentage of Participants with Sustained Virologic Response 12 Weeks Post-treatment (SVR12) in Arm A DB Active Drug Excluding Prior SOF + Ribavirin (RBV)	AbbVie	Phase 3	304	NCT02640482 (https://ClinicalTrials.gov/show/NCT02640482, Accessed on 22 March 2023)
2.	Treatment of Acute Hepatitis C Virus Infection with Pegylated Interferon in Injection Drug Users	Drug: Pegylated Interferon	The sustained viral response rate in the treatment group versus control (measured at Week 24), Adherence rate in the treatment group (measured at Week 24)	National Institute on Drug Abuse (NIDA), University of Washington	Phase 4	21	NCT00194480 (https://ClinicalTrials.gov/show/NCT00194480, Accessed on 22 March 2023)
3.	Drug Use & Infections in Vietnam - Hepatitis C (DRIVE-C)	Drug: Sofosbuvir 400 mg and Daclatasvir 60 mg, Drug: Sofosbuvir 400 mg and Daclatasvir 90 mg, Drug: Ribavirin, Drug: Sofosbuvir and Daclatasvir for 24 weeks	The proportion of all patients with the success of the model of care, the Proportion of patients with detectable HCV RNA	ANRS, Emerging Infectious Diseases	Phase 4	979	NCT03537196 (https://ClinicalTrials.gov/show/NCT03537196, Accessed on 22 March 2023)
4.	A Study to Evaluate the Efficacy and Safety of Three Experimental Drugs in Adults with Hepatitis C Virus Infection, Who Are Either Treatment-naive or Treatment-experienced in Brazil	Drug: ombitasvir/paritaprevir/ritonavir and dasabuvir, Drug: ribavirin	Percentage of Participants with Sustained Virologic Response 12 Weeks Post-treatment (SVR12), Change from Baseline to 12 Weeks After the Last Dose of Study Drug, (SF-36v2) Mental Component Summary (MCS) Scores: Change from Baseline to 12 Weeks After the Last Dose of Study Drug	AbbVie	Phase 3	222	NCT02442271 (https://ClinicalTrials.gov/show/NCT02442271, Accessed on 22 March 2023)
5.	Pegylated Interferon Plus Ribavirin in the Treatment of Active and Past Intravenous Drug Users Infected with Hepatitis C	Drug: pegylated interferon alfa-2a (Roche) and ribavirin	Sustained viral response	The University of Calgary, Canadian Institutes of Health Research (CIHR), Roche Pharma AG	Phase 4	66	NCT00203606 (https://ClinicalTrials.gov/show/NCT01773070, Accessed on 22 March 2023)
6.	A Follow-up Study Designed to Obtain Long-Term Data on Participants Who Either Achieved a Sustained Virologic Response or Did Not Achieve a Sustained Virologic Response in an AbbVie Sponsored Hepatitis C Study	Drug: ABT-450/ritonavir, Drug: ABT-333, Drug: ABT-267	Percentage of Participants Who Experienced Relapse-12 overall With and Without New HCV Infection	AbbVie (prior sponsor, Abbott), AbbVie	Phase 3	478	NCT01773070 (https://ClinicalTrials.gov/show/NCT01773070, Accessed on 22 March 2023)
7.	Pilot Treatment as Prevention for HCV Among Persons Who Actively Inject Drugs	Other: modified directly observed therapy (mDOT), Other: unobserved dosing, Other: Motivational Interviewing-based counseling	Number of people who inject drugs (PWIDs) with HCV who were recruited and retained, Medication adherence to study drug, Challenges of medication adherence, SVR (end-of-treatment response), SOF/metabolite levels, HCV relapse and reinfection, Social and injector networks of participants	Phillip Coffin, MD, MIA, National Institute on Drug Abuse (NIDA), San Francisco Department of Public Health	Phase 4	31	NCT02609893 (https://ClinicalTrials.gov/show/NCT02609893, Accessed on 22 March 2023)
8.	Hepatitis C Treatment in PWIDs: MAT or Syringe Exchange Assisted-therapy vs Standard of Care	Drug: elbasvir-grazoprevir (50 mg/100 mg)	SVR 12, SVR 48, Discontinuation Rate or Lost to Follow Up, NS5A Resistance, Medication Adherence, Injection Drug Use Relapse (IDU)	Oregon Health and Science University	Phase 4	100	NCT03093415 (https://ClinicalTrials.gov/show/NCT03093415, Accessed on 22 March 2023)
9.	A Trial to Reduce Hepatitis C Among Injection Drug Users - 1	Behavioral: Behavior Therapy	Hepatitis C seroconversion, Substance use	Butler Hospital, National Institute on Drug Abuse (NIDA)	Phase 3	277	NCT00218192 (https://ClinicalTrials.gov/show/NCT00218192, Accessed on 22 March 2023)
10.	A Study to Evaluate the Efficacy and Safety of Three Experimental Drugs Compared with Telaprevir (a Licensed Product) in People with Hepatitis C Virus Infection Who Have Not Had Treatment Before	Drug: ABT-450/r/ABT-267, ABT-333, Drug: Ribavirin, Drug: Telaprevir, Drug: Pegylated Interferon-alpha 2-a (PegIFN)	Percentage of Participants with Sustained Virologic Response 12 Weeks After Treatment (SVR12), Percentage of Participants with Sustained Virologic Response 24 Weeks After Treatment (SVR24)	AbbVie	Phase 3	311	NCT01854697 (https://ClinicalTrials.gov/show/NCT01854697, Accessed on 22 March 2023)
**(vi) HBV**							
1.	Effects of Telbivudine and Tenofovir Disoproxil Fumarate Treatment on the Hepatitis B Virus DNA Kinetics in CHB	Drug: Telbivudine, Drug: Tenofovir, Drug: Telbivudine plus tenofovir	Change in Hepatitis B Virus (HBV) Deoxyribonucleic Acid (DNA) Level from Baseline to Week 12		Phase 3	83	NCT00805675 (https://ClinicalTrials.gov/show/NCT00805675, Accessed on 22 March 2023)
2.	Effects of Telbivudine and Tenofovir Disoproxil Fumarate Treatment on the Hepatitis B Virus DNA Kinetics in CHB	Drug: Telbivudine, Drug: Tenofovir, Drug: Telbivudine plus tenofovir	Change in Hepatitis B Virus (HBV) Deoxyribonucleic Acid (DNA) Level from Baseline to Week 12		Phase 3	109	NCT00395018 (https://ClinicalTrials.gov/show/NCT00395018, Accessed on 22 March 2023)
3.	TDF VS LAM + ADV in LAM + ADV Treated LAM-resistant CHB Patients with Undetectable Hepatitis B Virus DNA	Drug: Lamivudine plus adefovir, Drug: Tenofovir	Percentage number of patients with virus reactivation, Virologic response, Antiviral resistance, Biochemical response, Serologic response, Safety assessment		Phase 4	171	NCT01732367 (https://ClinicalTrials.gov/show/NCT01732367, Accessed on 22 March 2023)
4.	Evaluation of Tenofovir Disoproxil Fumarate in Adolescents with Chronic Hepatitis B Infection	Drug: Tenofovir disoproxil fumarate (TDF), Drug: Placebo	Percentage of Participants with HBV DNA < 400 copies/mL at Week 72		Phase 3	106	NCT00734162 (https://ClinicalTrials.gov/show/NCT00734162, Accessed on 22 March 2023)
5.	A Study of Maraviroc In HIV Co-Infected Subjects With Hepatitis C and Hepatitis B	Drug: Maraviroc, Drug: Placebo	Percentage of Participants with grade 3 and grade 4 Alanine Aminotransferase (ALT) Abnormalities at Week 48		Phase 4	138	NCT01327547 (https://ClinicalTrials.gov/show/NCT01327547, Accessed on 22 March 2023)
6.	Viral Kinetics Study of Telbivudine and Entecavir in Adults with Chronic Hepatitis B	Drug: Entecavir, Drug: Telbivudine	Change in Mean Hepatitis B Virus (HBV) DNA Levels, Change in Mean HBV DNA Level, The Area Under the Curve (AUC) of HBV DNA Change, Change in Alanine Aminotransferase (ALT) Levels, Characterization of Very Early Viral Kinetics: Estimation of Viral Clearance, Characterization of Very Early Viral Kinetics: Estimation of the Rate of Infected Cell Loss, Characterization of Very Early Viral Kinetics: Estimation of the Efficiency Factor of Blocking Virus Production, Number of Patients Who Are Polymerase Chain Reaction (PCR) Negative		Phase 3	44	NCT00412529 (https://ClinicalTrials.gov/show/NCT03468907, Accessed on 22 March 2023)
**(vii) EBV**							
1.	Belatacept 3 Month Post Transplant Conversion Study	Drug: belatacept, Drug: Tacrolimus, Drug: MPA	Change in eGFR (MDRD) at 2 Years Post-transplant Compared to Baseline at Month 3 (Conversion), Acute Rejection, Graft Survival, Patient Survival	Lorenzo Gallon, Bristol-Myers Squibb, Northwestern University	Phase 4	28	NCT02213068 (https://ClinicalTrials.gov/show/NCT02213068, Accessed on 22 March 2023)
2.	A Trial of Adjuvant Chemotherapy in Nasopharyngeal Carcinoma Patients with Residual Epstein–Barr Virus (EBV) DNA Following Radiotherapy	Drug: Adjuvant chemotherapy (gemcitabine and cisplatin)	Relapse-free survival, Overall survival, Loco-regional control, Metastasis-free survival, Toxicity of adjuvant chemotherapy, Correlation of plasma EBV DNA and PET/CT scan with clinical course and outcome	Chinese University of Hong Kong, Hong Kong Nasopharyngeal Cancer Study Group Limited	Phase 3	104	NCT00370890 (https://ClinicalTrials.gov/show/NCT00370890, Accessed on 22 March 2023)
3.	A Phase III Trial Evaluating Chemotherapy and Immunotherapy for Advanced Nasopharyngeal Carcinoma (NPC) Patients	Biological: autologous EBV-specific Cytotoxic T cells, Drug: a combination of IV gemcitabine and IV carboplatin (AUC2)	Prolonging Overall Survival, Disease Progression, Overall Response Rate, Clinical Benefit Rate, and Quality of Life of patients	Tessa Therapeutics	Phase 3	330	NCT02578641 (https://ClinicalTrials.gov/show/NCT02578641, Accessed on 22 March 2023)
4.	Efficacy and Safety Study of Lenalidomide Plus R-CHOP Chemotherapy Versus Placebo Plus R-CHOP Chemotherapy in Untreated ABC Type Diffuse Large B-cell Lymphoma	Drug: lenalidomide, Drug: Placebo, Drug: Rituximab, Drug: Cyclophosphamide, Drug: Doxorubicin, Drug: prednisone, Drug: vincristine		Celgene	Phase 3	570	NCT02285062 (https://ClinicalTrials.gov/show/NCT02285062, Accessed on 22 March 2023)
5.	Newly Diagnosed Mature B-ALL, Burkitt’s Lymphoma and Other High-grade Lymphoma in Adults	Drug: Adriamycin, Drug: Cyclophosphamide, Drug: Cytarabine, Drug: Dexamethasone/Prednisolone, Drug: VP16, Drug: Ifosfamide, Drug: Methotrexate, Drug: G-CSF, Drug: Rituximab, Drug: Vincristine/Vindesine, Procedure: Irradiation (in specific conditions)		Nicola Goekbuget, Goethe University	Phase 4	650	NCT00199082 (https://ClinicalTrials.gov/show/NCT00199082, Accessed on 22 March 2023)
6.	Double Cord Versus Haploidentical (BMT CTN 1101)	Biological: Haploidentical Bone Marrow Transplant, Biological: Double Umbilical Cord Blood Transplant		Medical College of Wisconsin, National Heart, Lung, and Blood Institute (NHLBI), National Cancer Institute (NCI), Blood and Marrow Transplant Clinical Trials Network, National Marrow Donor Program	Phase 3	368	NCT01597778 (https://ClinicalTrials.gov/show/NCT01597778, Accessed on 22 March 2023)
7.	Interleukin-2 or Observation Following Radiation Therapy, Combination Chemotherapy, and Peripheral Stem Cell Transplantation in Treating Patients with Recurrent Non-Hodgkin’s Lymphoma	Biological: aldesleukin, Biological: filgrastim, Drug: cyclophosphamide, Drug: etoposide, Radiation: radiation therapy, Procedure: peripheral blood stem cell transplantation, Procedure: bone marrow ablation with stem cell support	Overall survival, Disease-free survival, Frequency, and severity of toxicity associated with post-transplant aldesleukin therapy	National Cancer Institute (NCI)	Phase 3	206	NCT00002649 (https://ClinicalTrials.gov/show/NCT01800071, Accessed on 22 March 2023)
8.	A Phase Ib Trial of MVA-EBNA1/LMP2 Vaccine in Nasopharyngeal Carcinoma	Drug: MVA-EBNA1/LMP2 vaccine	Immune response to three cycles of MVA-EBNA1/LMP2 vaccine, Occurrence of adverse events defined according to NCI CTCAE version 4.02, Immune memory, and recall response to MVA-EBNA1/LMP2 vaccination, Measurement of EBV genome levels in plasma before, during and after vaccination, Tumor response as determined by Immune-Related Response Criteria (irRC)	Cancer Research UK	Phase 1	22	NCT01800071 (https://ClinicalTrials.gov/show/NCT01800071, Accessed on 22 March 2023)
**(viii) HIV**							
1.	Prospective Evaluation of Etravirine for HIV-infected Patients in Need of Lipid-lowering Drugs	Drug: stop statin and switch to an antiretroviral drug with less impact on lipid metabolism	The proportion of patients not qualifying anymore for statin treatment, fasting lipids changes	Calmy Alexandra, Janssen-Cilag A.G., Switzerland, University Hospital, Geneva	Phase 3	34	NCT01543035 (https://ClinicalTrials.gov/show/NCT01543035, Accessed on 22 March 2023)
2.	Behavioral Drug and HIV Risk Reduction counselling in Methadone Patients in China	Behavioral: Behavioral Drug and HIV Risk Reduction Counselling (BDRC), Behavioral: Drug counselling	Reductions of illicit opiate use, Reductions in HIV risk behaviors	Yale University, National Institute on Drug Abuse (NIDA)	Phase 3	45	NCT00757744 (https://ClinicalTrials.gov/show/NCT00757744, Accessed on 22 March 2023)
3.	Positive Change Agents Program-Tanzania (Evaluation)	Behavioral: Appreciative Inquiry Change Agents (CA) program (NAMWEZA)	HIV Testing or other related services for network members of HIV Positive change agents, Depressive symptoms	Harvard School of Public Health (HSPH), Harvard Medical School (HMS and HSDM), Muhimbili University of Health and Allied Sciences, Centres for Disease Control and Prevention	Phase 3	1046	NCT01693458 (https://ClinicalTrials.gov/show/NCT01693458, Accessed on 22 March 2023)
4.	Safety and Efficacy of Switching from Regimens of ABC/3TC + a third Agent to E/C/F/TAF Fixed-Dose Combination (FDC) in Virologically Suppressed HIV 1 Infected Adults	Drug: E/C/F/TAF, Drug: ABC/3TC, Drug: Third Antiretroviral Agent	Percentage of Participants Who Have HIV1 RNA < 50 Copies/mL as Defined by the FDA Snapshot Algorithm at Week 24, Change from Baseline in CD4+ Cell Count at Week 48	Gilead Sciences	Phase 3	275	NCT02605954 (https://ClinicalTrials.gov/show/NCT02605954, Accessed on 22 March 2023)
5.	Using Drug Levels in the Blood to Guide Therapy in HIV Infected Patients Taking a Protease Inhibitor	Procedure: Therapeutic Drug Monitoring (TDM)	Change in log_10_ plasma HIV1 RNA concentration from Step 2 entry (Week 4) to Week 24 (20 weeks postrandomization), change in log_10_ plasma HIV1 RNA concentration from study entry to Week 24 (20 weeks post-randomization)	National Institute of Allergy and Infectious Diseases (NIAID)	Phase 3	360	NCT00041769 (https://ClinicalTrials.gov/show/NCT00041769, Accessed on 22 March 2023)
6.	Maraviroc is an Immunomodulatory Drug for Antiretroviral-treated HIV Infected Patients Exhibiting Immunologic Failure	Drug: Placebo, Drug: Maraviroc	Week 24 Change in Percentage of CD8+ T Cells That Co-express CD38 and HLA DR (Week 24 %CD38+HLA-DR+ CD8+ T Cells Minus Baseline %CD38+HLA-DR+ CD8+ T Cells), Change in CD4+ T Cell Count, Change in Ultrasensitive Plasma HIV RNA Level (Single Copy/mL Assay), Change in Brachial Artery Flow-mediated Dilatation (UCSF Site Only), Change in Gut-associated Lymphoid Tissue HIV RNA Level (UCSF Site Only)	University of California, San Francisco, Pfizer, amfAR, The Foundation for AIDS Research, Stanford University, Case Western Reserve University, Rush University	Phase 4	45	NCT00735072 (https://ClinicalTrials.gov/show/NCT00735072, Accessed on 22 March 2023)
7.	Anti-HIV Drug Regimens with or Without Protease Inhibitors and Drug Level Monitoring in HIV Infected Adolescents	Drug: Efavirenz + 2 NRTIs, Drug: Lopinavir/Ritonavir + 2 NRTIs, Procedure: Therapeutic Drug Monitoring	The proportion of patients achieving viral suppression (viral load less than 1,000 copies/mL) at Week 24 and maintaining suppression through Week 48 while remaining on study treatment, the proportion of patients achieving virologic suppression (viral load less than 1,000 copies/mL) at Week 24 and maintaining suppression through Week 96 while remaining on study treatment, CD4 (T helper cells), CD8 (cytotoxic T cells), naive CD4 T cells (CD62L/CD45RA/CD4), and activated CD8 T cells (HLA-DR/CD38/CD8), changes from baseline to Weeks 24, 48, and 96 for percentage and total number of CD19 (B cells), total T cells (CD3 T cells), CD4 (T helper cells), CD8 (cytotoxic T cells), naive CD4 T cells (CD62L/CD45RA/CD4), and activated CD8 T cells (HLA-DR/CD38/CD8)	National Institute of Allergy and Infectious Diseases (NIAID), Eunice Kennedy Shriver National Institute of Child Health and Human Development (NICHD)	Phase 3	240	NCT00075907 (https://ClinicalTrials.gov/show/NCT00075907, Accessed on 22 March 2023)
8.	Drug Interaction Study Between Lumefantrine and Lopinavir/Ritonavir	Drug: Lumefantrine - lopinavir/ritonavir drug interaction, Drug: Lumefantrine only arm		Makerere University, University of Liverpool	Phase 4	32	NCT00619944 (https://ClinicalTrials.gov/show/NCT00619944, Accessed on 22 March 2023)
9.	Antidepressant Medication for Reducing HIV Risk Behavior in Depressed Intravenous Drug Users	Drug: Antidepressant Medication	Maintenance of HIV risk-free drug behavior (measured at Month 12), Reduction in depressive symptoms (measured at Month 12)	Butler Hospital, National Institute of Mental Health (NIMH)	Phase 3	265	NCT00228007 (https://ClinicalTrials.gov/show/NCT00228007, Accessed on 22 March 2023)
10.	Drug Interaction Study of Famotidine and Atazanavir with Ritonavir in HIV-Infected Patients	Drug: Atazanavir/Ritonavir, Drug: Atazanavir/Ritonavir + Famotidine, Drug: Atazanavir/Ritonavir + Tenofovir Disoproxil Fumarate + Famotidine		Bristol-Myers Squibb	Phase 4	40	NCT00384904 (https://ClinicalTrials.gov/show/NCT00384904, Accessed on 22 March 2023)

a “Completed” status here means the study has ended and participants are no longer being examined or treated; that is, the last participant’s last visit has occurred.

## 5 Current Vaccines for Viral Oncogenesis Therapeutics

Over the past few decades, tumor immunotherapy research has advanced significantly, with many trials now being evaluated in clinical settings. Cancer vaccination is a potential treatment approach for immunizing tumor cells. Tumor antigens from cancer vaccines, which may be given as entire cells, peptides, nucleic acids, etc., induce antitumor immunity. Optimal cancer vaccines stimulate humoral and cellular immunity while overcoming tumor-induced immune suppression.^330^ Cancer vaccines vary from conventional vaccinations since their therapeutic goals involve using cellular immune responses specific to tumor antigens to destroy tumor cells. The hepatitis B virus and human papillomavirus are the only preventive vaccinations currently used to prevent virally induced cancers.^331^ Tumor antigens are also endogenous with limited immunogenicity in contrast to conventional vaccinations that contain antigens from foreign infections. It can be challenging to get the immune system to respond properly to tumor antigens.^332^ Traditional vaccinations also promote humoral immunity. Nevertheless, eliminating malignant cells for cancer vaccines depends on CD8+ cytotoxic T cell-mediated cellular immunity.^333^

Cancer vaccines fall into four categories, viz, nucleic acid-based vaccinations, viral-based vaccines, peptide-based vaccines, and cell-based vaccines. Initially, cancer vaccines take the form of cell-based vaccinations. Cell-based cancer vaccines elicit a larger immune response to antigens since they are frequently made from whole or fragmented cells that include tumor antigens. The dendritic cells (DC) vaccine is a crucial subset of cell-based vaccinations. Clinical trials of customized neoantigen cancer vaccines focused on DC have revealed positive antitumor outcomes. However, the creation of DC vaccines is constrained by laborious procedures and high expenses. Viral genetic material can be altered to include sequences containing tumor antigens since viruses are naturally immunogenic. Adenovirus is one of several recombinant viruses that can spread infection via immune cells.^330^

### 5.2.1 Mechanism of Cancer Vaccines

Optimal interactions between immunological and nonimmune components of the tumor microenvironment (TME) are necessary for effective antitumor immunity. Antigen-presenting cells (APCs), namely dendritic cells (DCs), capture and cross-present the antigens released by tumor cells and activate T cells.^334^ In the TME, natural killer (NK) cells, neutrophils, and macrophages of the innate immune system are extremely important for the immediate recognition and attack of tumor cells. When tumor cells undergo immunogenic cell death (ICD)—spontaneously or because of therapies such as some forms of chemotherapy—they emit molecular patterns linked with risk. As a result, DCs develop, take up, process, and present antigens on MHC class I (MHC-I) and MHC-II molecules (via antigen cross-presentation).^335^ These DCs go to secondary lymphoid organs where they bind MHC-T cell receptors and coreceptors to connect with naïve CD4+ T cells and CD8+ T cells. To prime T cells, migratory DCs also pick up antigens in the tumor microenvironment or the periphery and ″transfer″ them to lymph node-resident DCs.^336^ It is significant to highlight that the activation of CD8+ T cells and tumor immunity depend heavily on CD4+ T cell support. T cell stemness is becoming increasingly important as a regulator of tumor immunity and a determinant of the immunotherapy response. After cognate contacts, the activated T cells return to the TME to limit tumor development by direct killing and IFN-mediated inhibition of cancer cell proliferation.^337^

### 5.2.3 Current Vaccines and Their Effectiveness

Efforts toward vaccine development have been gaining immense attention. One of the vaccines, the gp350-Ferritin Nanoparticle Vaccine, has been developed for infection with the Epstein–Barr virus (EBV), which is linked to several human disorders, notably infectious mononucleosis (IM) as well as several types of cancers. Neoplasms following infection include stomach and nasopharyngeal carcinoma, Burkitt and Hodgkin lymphoma, and lymphoproliferative diseases. The likelihood that an EBV vaccination will improve public health is highlighted by the incidence and severity of these disorders.^338^ A murine monoclonal antibody (mAb) named 72A1 potently neutralizes EBV infections of B cells,^339^ and its anticipated epitope on gp350 closely resembles the anticipated binding site of CR2, pointing to a method of neutralization mediated by this antibody.^340^ According to Ogembo et al. (2013), the mAb 72A1 prevents gp350 from interacting with CR1, further demonstrating the functional importance of this epitope and making it a desirable vaccine target to stop viral infection of B cells, the primary target cell type of EBV.^341^ The researcher has taken gp350 into account to prevent EBV-linked disorders and thus developed a gp350-Ferritin Nanoparticle Vaccine.

Similarly, Interleukin 12 (IL-12) Vaccines have been developed because of the pivotal role of IL-12 in cancer immunotherapy. Interleukin 12 (IL-12) is a pleiotropic (affecting multiple unrelated organs) cytokine whose effects link to innate and adaptive immunity. IL-12 was initially identified as a substance released by B-cell lines that had undergone EBV transformation in response to PMA. IL-12 was initially known as the ″cytotoxic lymphocyte maturation factor″^342^ and ″natural killer cell stimulatory factor″^343^ based on its effects. IL-12 seems to be an excellent option for cancer immunotherapy in humans due to its ability to integrate both adaptive and innate immunity and potently stimulate the production of IFNs, a cytokine that coordinates natural processes of anticancer defense.^344^ However, the highly limited therapeutic efficacy of this cytokine and the severe adverse effects connected with the systemic injection of IL-12 in research studies significantly reduced excitement for its use in cancer patients.

Notwithstanding these hurdles, clinical oncology is still very interested in IL-12. The current review analyzes clinical trials focusing on ongoing investigations to increase the treatment effectiveness of IL-12 and reduce its toxicity. It also reviews the much more promising IL-12-based techniques in animal models.^345^ Moreover, IL-12 has been demonstrated to be highly efficient in animal models of tumor treatment due to its ability to promote various direct and indirect anticancer actions related to innate immunity, adaptive immunity, and nonimmune pathways. Many mouse models, including tumor cells and hematologic malignancies comprising low immunogenic tumors, have effectively used this.^345−350^ The anticancer effects of IL-12 have been amplified in several efforts. Combining IL-12 with numerous treatment modalities, including chemotherapeutics, cytokines, antibodies, antiangiogenic drugs, radiation, adoptive therapy, and tumor vaccines, can significantly increase its antitumor effectiveness.^345^

Many gene therapeutic techniques have been developed, permitting local and extended cytokine production to decrease IL-12-induced toxicities further and enhance its efficiency in experimental tumor treatment. The IL-12 gene has been inserted into a variety of viral^351−355^ and nonviral^356−358^ vectors, into developing tumors,^357,359−361^ or into fibroblasts designed to express IL-12 that have been injected at the location of an existing tumor.^345^ Vaccines containing tumor antigens,^362^ tumor cells,^351,352^ and dendritic cells^363−365^ have also been developed effectively using the IL-12 gene. Moreover, IL-12 has improved the anti-cancer effects of adoptive therapies using IL-12-secreting specific T cells^366^ or Herpes simplex virus.^367^ Many vaccines have been developed against oncogenic viruses for potential therapeutics, summarized in Table 4.

**Table 4:** Available Potential Vaccines against Oncogenic Viruses

S. No.	Viruses	Vaccines	References
1.	Hepatitis B virus (HBV)	Engerix-B and Recombivax HBHEPLISAV-B	(368)
2.	Human Papilloma Virus (HPV)	Gardasil (a bivalent HPV vaccine), Cervarix	(368)
3.	Kaposi sarcoma herpesvirus (KSHV/HHV-8)	Epitopes-based vaccines mRNA-based vaccines (Potential candidates)	(369,370)
4.	Epstein–Barr virus (EBV/HHV-4)	EBV gp350-Ferritin nanoparticle vaccine (Under trials)	(371)
5.	Merkel cell polyomavirus (MCPyV)	A VP1-target vaccine formulated with CRA (Potential candidate), Interleukin-12 (IL-12) Plasmid Vaccines	(372,373,374)
6.	Hepatitis C virus (HCV)	New Hepatitis C Prophylactic Vaccine (Under trials)	(375)
7.	Human T-cell leukemia/ Lymphotropic virus-1 (HTLV-1)	TAX protein-based epitopes vaccine peptide-based vaccine (HBZ peptide) (Potential candidates)	(376,377)
8.	Human immunodeficiency virus (HIV)	BG505 MD39.3, BG505 MD39.3 gp151, and BG505 MD39.3 gp151 CD4KO HIV Trimer mRNA Vaccines	(378)

## 6 Challenges Associated with Antivirals during Viral Oncogenesis

The interplay of certain oncoviruses and the onset of cancer has become definitive in recent times. With an approximate value of 15–20% of various human cancers being caused by such oncoviruses, it has raised a medical concern all over the globe. The onset of cancer cannot be concluded on the basis of single or limited aspects *indeed many factors could be involved.^3,89^ The general mechanism followed by cancer-causing viruses was reported to be mediated by disrupting the tumor suppressor gene, p53, and retinoblastoma, pRb.^8^ Scientists have been on the verge of developing treatment modalities to treat such virus-induced oncogenesis. Antivirals are considered to be the most common treatment modality to treat viral infections. Still, the rapidly changing and evolving nature of viruses makes it challenging to develop antivirals that could be effective against viral oncogenesis every time.^379^

Antiviral therapy for EBV-induced tumors includes acyclovir, famciclovir, and ganciclovir, which play potential inhibitory role against EBV replication during the lytic phase.^249^ The main mechanism behind the functioning of EBV-based antivirals depends on the activation of lytic phase-associated kinases, which convert the antiviral’s inactive form to its active form. With this conversion, the level of viral replication is lowered, eventually destroying virus-induced tumor cells. The challenge behind this strategy can be seen during the lytic phase induction of EBV.^380^ Further the induction poses a high risk of circulation and distribution of viral particles within the host.^381^

Similarly, antiviral therapy for HHV-8-induced sarcoma, includes Ganciclovir and Valganciclovir, which plays an important role in the inhibition of HHV-8 replication in a trial encompassing 26 HHV-8-infected patients.^382,383^ With the eventual administration of ganciclovir, the serum levels of HHV-8 were reported to get reduced. However, recent clinical studies using valganciclovir showed that the potential of inhibition seems to have reduced.^384^ Cidofovir administration showed no reduction in HHV-8 viral loads in the plasma; thus, it may not show clear evidence about the effect of antivirals concerning HHV-8-associated infection.^385^ In recent times, siRNA-based antivirals have gained a great deal of importance. Studies have documented the potent knockdown potential of siRNA against disease-associated factors.^386^ siRNA is potent against HBV, HPV, and HCV.^387^ However, certain shortcomings are found in current research, such as runoff of virus, inadequate uptake by cells, reduced stability, adverse effects around nonspecific targets, and immune levels elevation.^388^ Compounds such as lamivudine, adefovir, emtricitabine, entecavir, and telbivudine are potent inhibitors of HBV replication in cell lines. Toxic effects on lactic acid levels and metabolism, severe liver illness, and, in rare cases, genotypic and phenotypic resistance exhibited by the virus strain after treatment halted further development of such drugs.^389^ Although there are no medicines available for selective antiviral for HPV, however, several nonselective methods are in use.^390^ Moreover, DNA helicase, the sole molecular target, may seem promising for inhibitor development, even though it has been found difficult to produce and establish in experiments. The viral polymerase NS5b, protease NS3/4a, and helicase NS3 are attractive targets in HCV. The virus internal ribosome entry site (IRES), antisense inhibition, and the NS2 zinc-dependent protease have limited targets that might be studied for improved treatment outcomes. Despite the potency of NNRTI inhibitors of HCV polymerase, may block a fraction of the six HCV strains, thus limiting their usefulness.^391,392^ There are three types of viral polymerase inhibitors used to treat HIV infection: (i) nucleoside reverse transcriptase inhibitors (NRTIs) (such as zidovudine, lamivudine, and abacavir), (ii) nucleotide reverse transcriptase inhibitors (NtRTIs), (of which tenofovir is the only approved one) and (iii) non-nucleoside reverse transcriptase inhibitors (NNRTIs). HIV inhibitors, whether NRTIs, NNRTIs, and protease inhibitors (PIs), or fusion inhibitors (FIs) (like Enfuvirtide), have had their efficacy severely curtailed by the rapid development of resistance and unwanted side effects.^391,392^

Resistance development and toxin generation are likely constrained by other viral processes such as fusion, viral coreceptor, and integration.^393^ It is tempting to speculate that certain anti-HIV1 medications targeting HIV1 RT would also be efficacious against HTLV1 RT because RNA reverse transcription is required to replicate both HIV1 and HTLV1 retroviruses. Both RTs are closely linked with the evolutionary tree. Indeed, it was proven in cell-to-cell transmission studies that AZT, 3TC, carbocyclic phosphonate 2′-oxa-3′-aza-nucleoside, and tenofovir inhibited HTLV1 transmission.^394−397^ Although, in recent findings, no immunological or clinical responses were seen other than some modest reductions in HTLV1 PVL were observed.^184^ In addition, further evidence revealed that AZT, regardless of the viral conditions of treated cells, might block telomerase and lead to cell death. The fact that HTLV1 is transmitted almost exclusively through cell-to-cell contact may further reduce the effectiveness of AZT and other NRTIs in combating the virus.^184^ Adefovir dipivoxil and tenofovir disoproxil are significantly more effective than AZT in reducing HTLV1 cell-to-cell transmission.

## 7 Future Perspectives and Concluding Remarks

A viral infection heavily influences the global cancer burden, and these viruses are becoming a leading cause of death and life-threatening diseases.^398^ Indeed, human tumor virology has attracted a great deal of interest in recent years. Oncogenic viruses have used various strategies to hijack the cellular pathways required for carcinogenesis. With the advent of technologies and systems-level studies, the concepts and practice of cancer treatment prevention have been revolutionized. For some oncogenic viruses, there is still a lack of convenient, effective antiviral agents that can be used for primary prevention. Therefore, despite the advances in delivering effective antiviral therapies, the currently available antiviral medications have many concerns, including high costs, drug resistance, safety concerns, and efficacy limits.^398,399^

Translation inhibitors have shown some success in targeting cancer cells. It is profitable to create drugs targeting many translation-related proteins. The challenge lies in targeting these inhibitors precisely to cancer cells. The vast array of proteins at play will provide drug designers with endless opportunities for targeted therapy.^400−402^ Researchers have developed some highly specific, safe, and effective cancer therapies that inhibit translation; this field of inhibitor development will likely continue for a long time due to newly discovered targets and our ever-increasing knowledge of the biological basics of these processes.

Nature has been a vital source of medical products and novel antiviral agents for ages. Studies have shown that secondary metabolites derived from various insects, marine organisms, microbes, and medicinal plants effectively prevent cancer patients infected with a virus.^403−405^ In contrast to combinatorial synthesized compounds, these natural bioactive compounds show excellent biochemical specificity against a wide range of molecular targets, while being absorbed and digested with minimal toxicity. Furthermore, it is well known that eating a diet rich in antioxidants has health benefits. Cancer therapies like chemotherapy and radiation therapy have various adverse effects, necessitating complementary medicine to treat cancer.^406−409^ Nowadays, two or more cancer therapies are usually administered to reduce the risk of acquiring resistance. Immunotherapy and oncological viral therapies, which use the patient’s immune system to attack cancer cells, are the latest additions to cancer treatment strategies. With advanced techniques, future research should be focused on efficiently separating and identifying valuable bioactive components from the chemically diversified natural product extracts, which can be employed in drug development. Moreover, these natural substances can be recommended to rural and underprivileged people to cure cancers since they are less costly and have virtually no side effects.^406−409^

In conclusion, future research activities should continually improve the understanding of the relationship between the dynamics of viruses and the natural history of diseases. These efforts could add to our arsenal of antivirals against these viruses and yield promising outcomes in adjuvant therapy for viral oncogenesis. Additionally, the failure of drugs in human trials is a general phenomenon that must be examined and addressed.

## Data Availability

Not Applicable.
